# Uroplakin traffic through the Golgi apparatus induces its fragmentation: new insights from novel in vitro models

**DOI:** 10.1038/s41598-017-13103-x

**Published:** 2017-10-09

**Authors:** Tanja Višnjar, Giancarlo Chesi, Simona Iacobacci, Elena Polishchuk, Nataša Resnik, Horst Robenek, Marko Kreft, Rok Romih, Roman Polishchuk, Mateja Erdani Kreft

**Affiliations:** 1 Institute of Cell Biology, Faculty of Medicine, University of Ljubljana, Vrazov trg 2, SI-1000 Ljubljana, Slovenia; 2 Telethon Institute of Genetics and Medicine (TIGEM), Via Campi Flegrei 34, 80078 Pozzuoli, (NA) Italy; 3 Institute for experimental musculoskeletal medicine, University of Münster, Albert-Schweitzer-Campus 1, Domagkstrasse 3, 48149 Münster, Germany; 4 Department of Biology, Biotechnical Faculty, University of Ljubljana, Večna pot 111, Ljubljana, Slovenia & LN-MCP, Institute of Pathophysiology, Faculty of Medicine, University of Ljubljana & Celica Biomedical Center, Ljubljana, Slovenia

## Abstract

Uroplakins (UPs) play an essential role in maintaining an effective urothelial permeability barrier at the level of superficial urothelial cell (UC) layer. Although the organization of UPs in the apical plasma membrane (PM) of UCs is well known, their transport in UCs is only partially understood. Here, we dissected trafficking of UPs and its differentiation-dependent impact on Golgi apparatus (GA) architecture. We demonstrated that individual subunits UPIb and UPIIIa are capable of trafficking from the endoplasmic reticulum to the GA in UCs. Moreover, UPIb, UPIIIa or UPIb/UPIIIa expressing UCs revealed fragmentation and peripheral redistribution of Golgi-units. Notably, expression of UPIb or UPIb/UPIIIa triggered similar GA fragmentation in MDCK and HeLa cells that do not express UPs endogenously. The colocalization analysis of UPIb/UPIIIa-EGFP and COPI, COPII or clathrin suggested that UPs follow constitutively the post-Golgi route to the apical PM. Depolymerisation of microtubules leads to complete blockade of the UPIb/UPIIIa-EGFP post-Golgi transport, while disassembly of actin filaments shows significantly reduced delivery of UPIb/UPIIIa-EGFP to the PM. Our findings show the significant effect of the UPs expression on the GA fragmentation, which enables secretory Golgi-outpost to be distributed as close as possible to the sites of cargo delivery at the PM.

## Introduction

Plasma membrane proteins must be correctly synthesized, processed and transported to the plasma membrane (PM) in order to perform their specialized function. Four major transmembrane proteins, the uroplakins (UPs), i.e., UPIa (27 kDa), UPIb (28 kDa), UPII (15 kDa) and UPIIIa (47 kDa)^1–5^ are expressed in a differentiation-dependent manner^2,6^ and are highly organized in so called urothelial plaques in the apical PM of highly differentiated superficial urothelial cells (UCs)^7,8^. When they are correctly assembled in the apical PM they provide the structural basis for the blood-urine barrier in the urinary bladder. Recently, it was shown that loss of UPIb results in urothelial plaque disruption in the bladder^9^. Moreover, the fact that no truncation or frame shift mutations of uroplakins have been found in any of primary vesicoureteral reflux (VUR) patients and that some breeding pairs of UPIII knockout mice yield litters that show not only small urothelial plaques, urothelial leakage and VUR, but also severe hydronephrosis and neonatal death, raises the possibility that major uroplakin mutations could be embryonically or postnatally lethal in humans^10–12^. Although the organization of UPs in the apical PM of UCs is well known, the biosynthetic pathway of UPs and their transport in UCs is still not completely understood.

Various studies examining UP transport predict a model of UP synthesis and their assembly into urothelial plaques. Based on this model UPs are synthesized in the ER where they must form two types of heterodimers (UPIa/UPII and UPIb/UPIIIa) before they can exit the ER^13^. UP-heterodimers are probably transported from the ER to the Golgi apparatus (GA), since UPIb isolated from mouse and human urothelial plaques, and UPIIIa isolated from mouse, cattle and human urothelial plaques contain complex glycans, which are added to the proteins in the GA^14–16^. The involvement of the GA in the modification of UPs is supported also by the observation that the prosequence of UPII can be cleaved by the GA-protease furin^17^. Sugar modifications and conformational changes of UPs probably induce the formation of a heterotetramer (UPIa/UPII-UPIb/UPIIIa). Six heterotetramers assemble into 16-nm uroplakin particle^7,18^. In post-Golgi vesicular compartments these 16-nm UP particles gradually arrange into semi-crystalline urothelial plaques^19,20^. Indeed first descriptions of the urothelial plaque structure in trans GA network are dating back to the 70s^21,22^, when first indication of GA contribution in UP biosynthetic pathway was shown in rat urothelium^23^ and urothelial plaque structures were shown in the GA by freeze-fracturing^21,22^. Freeze-fracture images disclosed post-Golgi vesicular compartments, namely UP-positive discoidal or fusiform-shaped vesicles (DFVs) in close association with the GA and the apical PM. Since the size of urothelial plaques on the membrane of DFVs resemble those found in close proximity to larger ones in the apical PM, it is believed that these associations are ideally configured to function in the intracellular synthesis and transport as well as the cytoplasmic-plasmalemmal transfer and the progressive incorporation of UPs into urothelial plaques in the apical PM^24^. Additional insights into the formation of urothelial plaques, i.e. their gradual aggregation or segregation in the apical PM of superficial UCs were shown from a combination of various microscopic techniques^8^. All these results therefore predicted the classical ER-GA pathway of UP biosynthesis. However, UPs have never been demonstrated in the GA, which opens the possibility that UPs could also bypass the GA. Supporting this hypothesis is the finding that UPIa and occasionally UPIb isolated from the plaques contain high-mannose residues added in the ER^14^, while in theory these residues should be removed from the proteins only in the GA.

We have shown previously that the GA undergoes major structural rearrangement during UC differentiation *in vivo* and *in vitro* ^25,26^. In this process the GA changes from a small ribbon like form which is positioned close to the nucleus into a large fragmented form that is spread throughout the cytoplasm of superficial UCs. Because increased synthesis of UPs coincides with the fragmentation of the GA, it is proposed that fragmentation of the GA and its redistribution to the cell periphery represent one of the key events that promote the uniform delivery of UPs to the apical PM^26^. Although our data suggested that the GA has a central role in the biosynthetic pathway of UP transport, neither the mechanisms nor the reason(s) of GA fragmentation are known.

In the transport of UPs from the GA to the apical PM the cytoskeleton plays an important, but not fully understood role. For example, it was shown that cytokeratins form a specific network that may regulate the transport of UPs-delivering vesicles to the apical PM^27,28^. Interestingly, actin filaments (AFs) are greatly retracted from the subapical cytoplasm of terminally differentiated superficial UCs^29^ and also microtubules (MTs) are rearranged during the differentiation of UCs^26,30^.

In this study, we generated specific UP cDNA constructs to follow the biosynthetic pathway of UPIb and UPIIIa, and examined the dynamics of UPIb/UPIIIa transport in living UCs. Using cell transfection, we also studied biosynthesis and transport of UPIb/UPIIIa in MDCK and HeLa cells, which do not synthesize UPs endogenously. With the aid of time-lapse microscopy, immunohistochemistry and electron microscopy (EM), we showed that transport of UPIb or UPIb/UPIIIa through the GA cause fragmentation and redistribution of the GA both in UCs and in cells that do not express UPs endogenously. Furthermore, the involvement of MTs and AFs in UP transport to the PM was revealed. Our findings elucidate how UCs adopted the biosynthetic pathway and transport mechanisms to ensure the assembly and delivery of their specific cargo – namely UPs - to the apical PM.

## Results

### Normal porcine UC cultures synthesize UPs and form urothelial plaques

**Figure 1:**
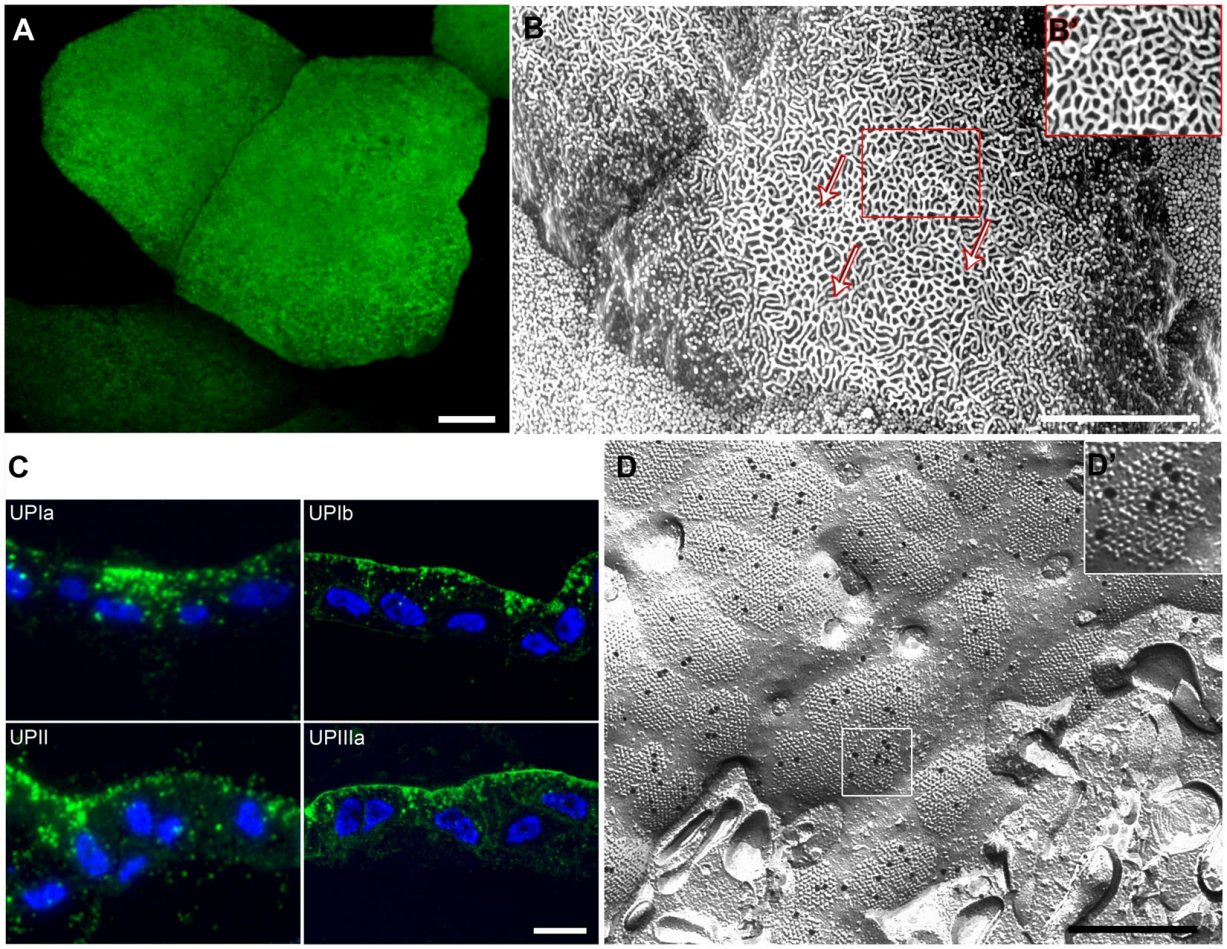
UPs in an apical PM of normal porcine UCs *in vitro*. (**A**) Immunofluorescence labeling of the 2-month urothelial model with anti-UPs shows large superficial UCs with expressed UPs (green). (**B**) Scanning electron microscopy shows an apical PM of an UC mostly shaped into rounded ridges (arrows and further enlarged in B’). (B’) Area in smaller red frame is increased by 50%. (**C**) Immunofluorescence labeling of semi-thin cryosections with anti-UPIa, anti-UPIb, anti-UPII and anti-UPIIIa shows positive signals of all four UPs in the apical PM and in cytoplasmic vesicles of superficial UCs. (**D**) FRIL. The normal porcine UC *in vitro* with UP-positive urothelial plaques in the apical PM. The immunogold labelling of UPs (black dots) is seen on the E-face within the urothelial plaques. (D’) Area, which corresponds to white frame in D, is increased by 100%. Bars: 10 µm (**A**–**C**), 500 nm (**D**).

To achieve highly differentiated UCs, we grew normal porcine UCs first in medium with low Ca^2+^ concentration and added serum for 7 days, which stimulates proliferation and leads to confluence, and then in medium with high Ca^2+^ concentration and without serum for 60 days that leads to stratification and differentiation^31,32^. First, RT-qPCR analysis of individual subunits UPIb and UPIIIa confirmed their expression in highly differentiated UCs *in vitro* (see Supplementary Fig. S1). Next, we therefore analysed UP expression at the protein level and UP cellular localization. Immunofluorescence labeling with a rabbit polyclonal antibody against all four UPs (anti-UPs)^1^ showed UP-positive apical PM of superficial UCs in an established three-to-five-layered urothelial model (Fig. 1A). Scanning EM analysis of the cell surface topography revealed an apical PM of superficial UCs mainly shaped in rounded ridges and rarely in microvilli (Fig. 1B), which is all in line with our previously published results^32,33^. In addition, the immunofluorescence labeling of cryo semi-thin sections with antibodies against UPIa, UPIb, UPII and UPIIIa showed positive signals of all four UPs in superficial UCs (Fig. 1C). All four UPs in the apical PM of UCs were additionally confirmed by the freeze-fracture replica immunolabeling (FRIL) technique (Fig. 1D). FRIL showed that anti-UPs antibodies recognized intramembrane particles that most likely represent 16-nm UP particles, which were organized into urothelial plaques (Fig. 1D). Between plaques there were areas devoid of UP particles, probably representing so called hinge regions. These results demonstrated that endogenously expressed UPs are not only targeted to the apical PM of normal porcine UCs *in vitro*, but also form urothelial plaques in these cells, which is novel.

Together our data show that normal porcine UCs in culture reach a high differentiation stage that is comparable to the differentiation stage of superficial UCs of normal urothelium *in vivo* in various species^34–36^ and therefore can be used as a suitable model for studies of UP transport.

### Individual EGFP-tagged UP subunits exhibit different ability to reach the apical PM of polarized UCs or MDCK cells

**Figure 2:**
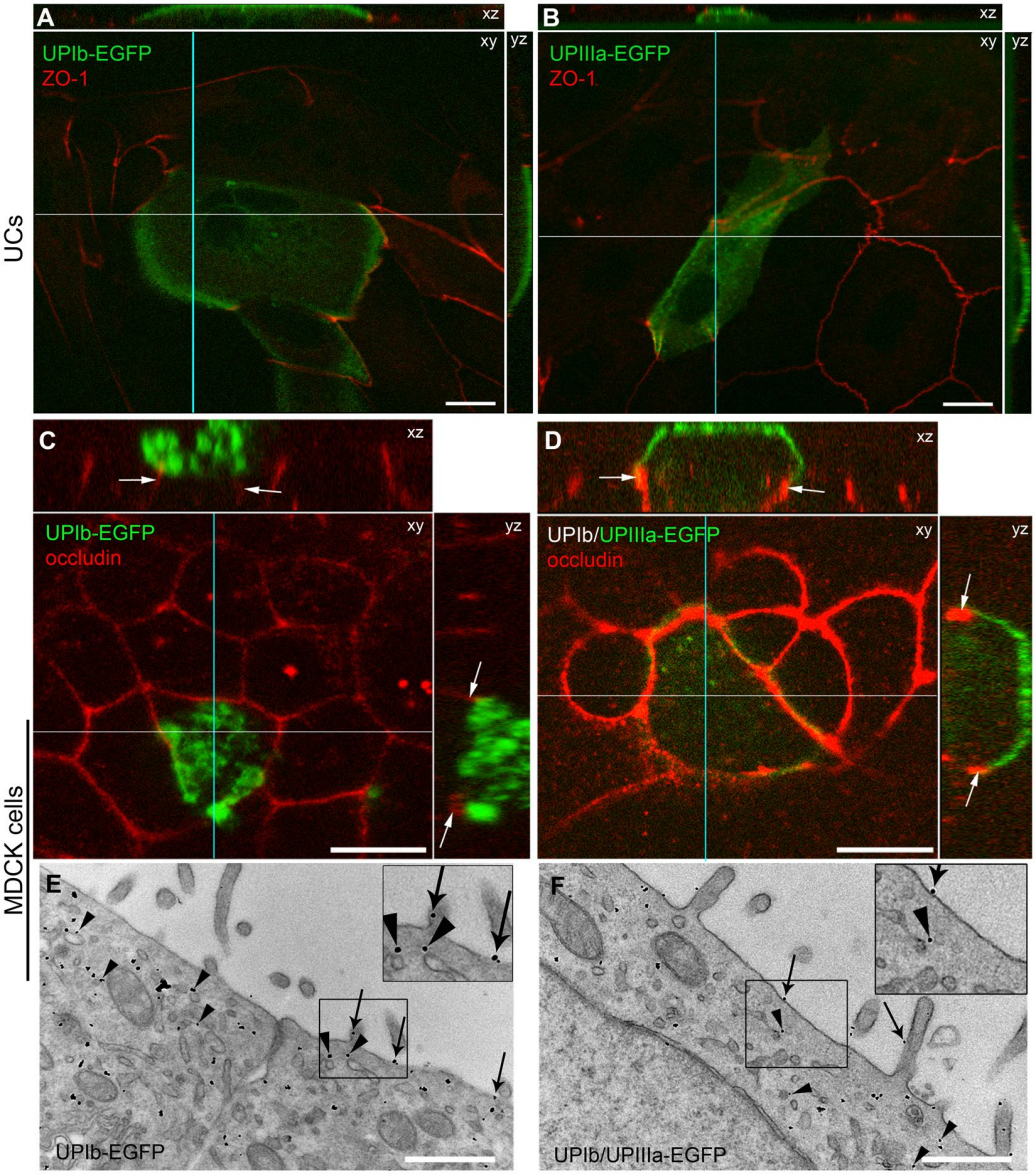
UPIb-EGFP and UPIIIa-EGFP are transported to the apical PM of polarized UCs and MDCK cells. (**A**,**B**) UCs were transfected with (**A**) UPIb-EGFP or (**B**) UPIIIa-EGFP and after one week of culturing stained with the antibody against ZO-1 (red). Confocal optical sections revealed that UPIb-EGFP and UPIIIa-EGFP were successfully transported to the apical PM (green) of superficial UCs. (**C**–**F**) Polarized MDCK cells transfected with (**C**) UPIb-EGFP or (**D**) co-transfected with UPIb/UPIIIa-EGFP and stained with the antibody against occludin (red). Z-stacks revealed that UPIb-EGFP and UPIb/UPIIIa-EGFP are located above the occludin (arrows) in the apical PM (green). (**E**,**F**) Transmission EM of polarized MDCK cells transfected with (**E**) UPIb-EGFP and (**F**) UPIb/UPIIIa-EGFP. Immunoelectron microscopy shows (**E**) UPIb-EGFP and (**F**) UPIb/UPIIIa-EGFP at the apical PM (arrows) and in vesicles (arrowheads) under the apical PM in E and F. (**E**,**F**) Area, which corresponds to black frame in (**E** and **F**), is increased by 50%. Bars: 10 µm (**A**–**D**), 500 nm (**E**,**F**).

First we tested whether EGFP–tagged UPIb and UPIIIa arrive at the apical PM in the same way as endogenous UPIb and UPIIIa in UCs. After transfection of UCs with UPIb-EGFP and UPIIIa-EGFP, the signal of UPIb-EGFP and UPIIIa-EGFP was located at the apical PM (Fig. 2A,B). These observations confirmed that the assembly, sorting and targeting of UPs to the apical PM of polarized UCs are not affected by the attached EGFP to the cytoplasmic tail of UPIb (i.e. UPIb-EGFP) and UPIIIa (i.e. UPIIIa-EGFP). Next, we wanted to confirm that transport of EGFP-tagged UPIb and UPIIIa to the apical PM can also be studied in other cell lines, like polarized epithelial MDCK cells. We transfected MDCK cells with UPIb-EGFP, UPIIIa-EGFP and UPIb/UPIIIa-EGFP (see Supplementary Tables S1 and S2). Confocal images revealed UPIb-EGFP signal in the apical PM above the immunolabelled tight junctional protein occludin (Fig. 2C). In contrast, when UPIIIa-EGFP was expressed alone, its signal overlapped with the signal of ER Tracker (see Supplementary Fig. S2). UPIIIa-EGFP signal was observed in vesicles and at the apical PM when co-expressed with its partner UPIb (Fig. 2D). To further confirm correct targeting of UPIb-EGFP and UPIb/UPIIIa-EGFP to the apical PM, we performed immunoelectron microscopy. Immunolabeling of EGFP confirmed the presence of UPIb-EGFP and UPIb/UPIIIa-EGFP in the apical PM of polarized MDCK cells (Fig. 2E,F).

Together, our results indicated that EGFP-tag does not interfere with the transport of UPIb and UPIIIa to the apical PM of polarized UCs and MDCK cells.

### Transport of UPs through the GA causes its fragmentation in different cell types

We have shown previously that the GA undergoes fragmentation during differentiation of UCs^26^, which probably facilitates the delivery of UPs over the entire apical PM of differentiating UCs. Here we further investigated the possible mechanisms and the reasons of GA fragmentation. Our aims were to find out (**1**) whether UPIb and UPIIIa passes the GA, (**2**) if GA fragmentation is intrinsic to UCs, (**3**) whether UP synthesis and transport are involved in fragmentation and redistribution of the GA, and (**4**) whether UPs cause fragmentation of the GA through induction of apoptosis.

**Figure 3:**
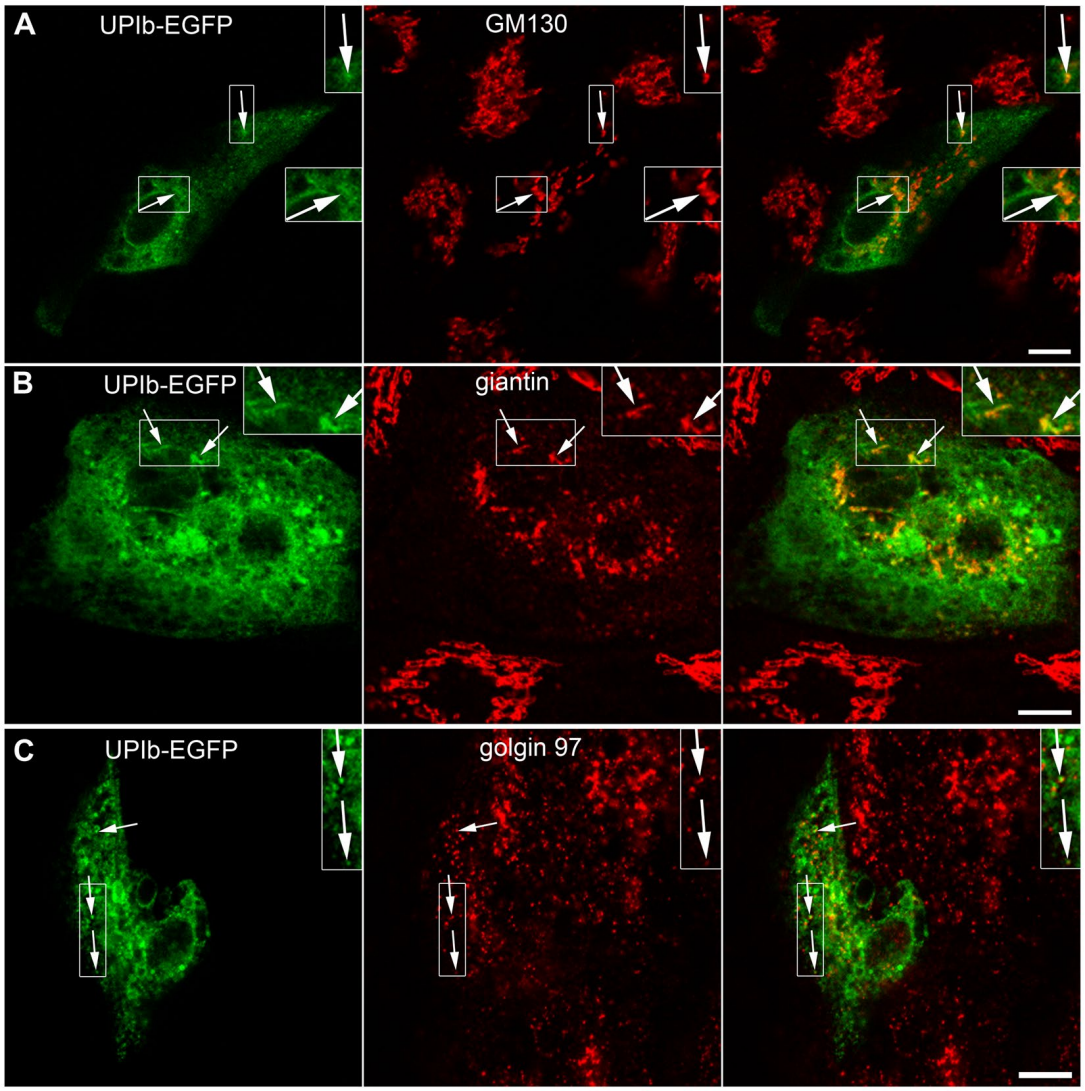
Expression and localization of UPIb-EGFP in non-polarized UCs. Immunofluorescence labelling of (**A**) GM130 (cis-GA marker); (**B**) giantin (cis- and medial GA marker) and (**C**) golgin-97 (trans-GA network marker) in UPIb-EGFP expressing UCs shows the presence of UPs in different parts of the GA. (**A**–**C**) Arrows show the green signals (UPIb-EGFP), red signals (GM130, giantin, golgin 97) and overlap of green and red signals (merged). Areas in larger frames are 50% increased smaller frames. Bars: 10 µm (**A**–**C**).

**Figure 4:**
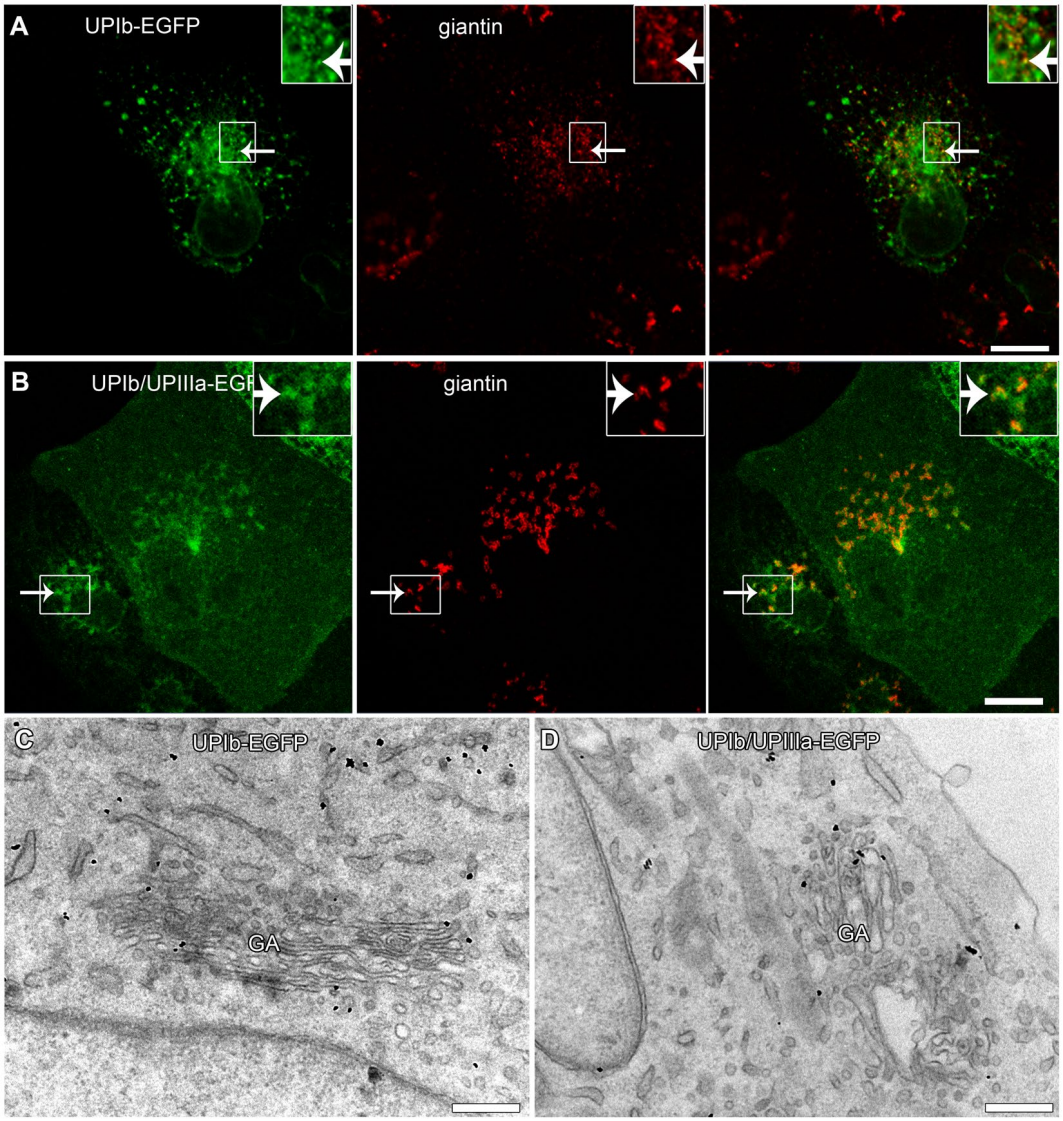
Expression and localization of UPIb-EGFP and UPIb/UPIIIa-EGFP in non-polarized MDCK cells. MDCK cells were transfected with (**A**) UPIb-EGFP or (**B**) co-transfected with UPIb/UPIIIa-EGFP and labelled with the antibody against giantin. (**A**,**B**) Both, UPIb-EGFP and UPIb/UPIIIa-EGFP signals overlap with the GA marker giantin (arrows). Smaller frames are 100% enlarged insets on the right. (**C**,**D**) The presence of UPIb-EGFP (black dots in **C**) and UPIb/UPIIIa-EGFP (black dots in **D**) in the GA is seen also at the ultrastructural level. Bars: 10 µm (**A**,**B**); 500 nm (**C**); 1 µm (**D**).

First, fluorescence labelling with ER and GA markers showed that UPIb-EGFP and UPIb/UPIIIa-EGFP colocalize with ER (Supplementary Fig. S2) and markers of GA (GM130, giantin and golgin 97) in UCs (Fig. 3) and also in MDCK cells (Fig. 4A,B). The presence of UPIb-EGFP and UPIb/UPIIIa-EGFP in the GA was further confirmed by immunoelectron microscopy (Fig. 4C,D). Moreover, in UCs also UPIIIa-EGFP colocalized with ER and GA (data not shown), probably due to its endogenously expressed partner UPIb. These results prove that UPIb and UPIb/UPIIIa are transported via the classical ER-GA pathway as was proposed in previous studies^17,37^.

**Figure 5:**
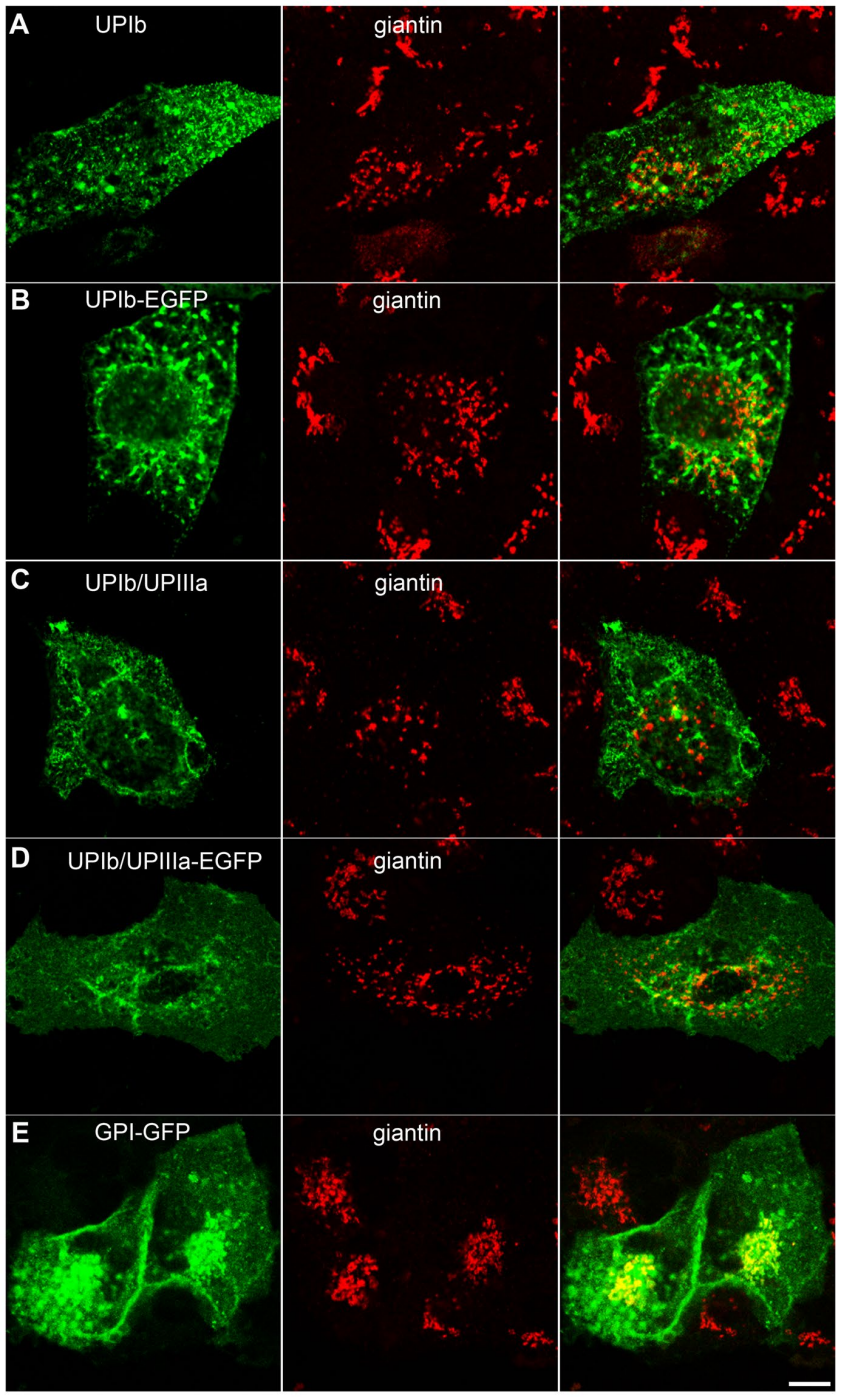
GA structure in non-polarized MDCK cells transfected with (**A**) UPIb; (**B**) UPIb-EGFP, (**C**) UPIb/UPIIIa, (**D**) UPIb/UPIIIa-EGFP, or (**E**) GPI–GFP. Cells transfected with UPIb were immunolabelled with antibodies against UPIb and cells transfected with UPIb/UPIIIa were immunolabelled with antibodies against UPIIIa. (**A**,**B**) Anti-giantin immunolabeling shows that transport of untagged or EGFP-tagged UPIb causes fragmentation of the GA. Note untagged and tagged UPIb on the PM of MDCK cells. (**C**,**D**) The transport of assembled heterodimers UPIb/UPIIIa or UPIb/UPIIIa-EGFP also causes fragmentation of the GA. Untagged and tagged UPIb/UPIIIa heterodimers are seen on the PM of MDCK cells. (**E**) The GA structure is not changed in GPI-GFP-expressing MDCK cells. Bars: 10 µm (**A**–**E**).

Second, we analyzed the GA architecture in UP-expressing and control (non-transfected) MDCK and HeLa cells. We found that fragmentation of the GA is not intrinsic only to UCs, but also to cells transfected with UP cDNA constructs (see Supplementary Tables S1 and S2). UPIb, UPIb-EGFP, UPIb/UPIIIa, or UPIb/UPIIIa-EGFP transfected MDCK and HeLa cells exhibited increased fragmentation of the GA in comparison to the control cells and in comparison to the cells expressing other GFP-tagged apical protein, GPI-GFP (Fig. 5 and Supplementary Figs S3 and S4). The analysis of untagged and EGFP-tagged UPs showed that EGFP tags do not increase the number of GA fragments (see Supplementary Fig. S4), while the expression of EGFP alone did not change the GA structure (data not shown). At the beginning of synthesis, when UPIb-, UPIIIa-, and UPIb/UPIIIa-EGFP still remain in the ER, the GA does not exhibit significant fragmentation (see Supplementary Table S3).

Third, the first changes in GA structure were only observed when UPs were detected within the GA, post-Golgi carriers/vesicles or at the PM (see Supplementary Table S3). Results of quantitative analyses of GA fragmentation are shown in Supplementary Fig. S4. The average number of GA fragments in UPIb-EGFP transfected MDCK cells was 60 ± 2.9 (s.e.m.; n = 10) and in HeLa cells it was 83.1 ± 6.9 (s.e.m.; n = 10). Similar, the average number of GA fragments in UPIb/UPIIIa transfected MDCK cells was 32.6 ± 4.4 (s.e.m.; n = 10) and HeLa cells was 45.8 ± 5.9 (s.e.m.; n = 10). This was statistically higher than the number of GA fragments in control non-transfected cells (7 ± 1 fragments (s.e.m.; n = 10) in MDCK; and 2.9 ± 0.5 (s.e.m.; n = 10) in HeLa cells) (see Supplementary Fig. S4A). To measure the extent of the GA fragmentation, we calculated the Euclidean distance, which determines the distance between two points. We observed increased Euclidean distance between GA fragments in UP-expressing cells in comparison with the control MDCK and HeLa cells (see Supplementary Fig. S4B). These results are in accordance with the increased number of GA fragments and Euclidean distance in differentiating UP-expressing UCs^26^. Moreover, a cluster analysis revealed that transfection with UP cDNA constructs did not affect significantly the number of GA fragments per cluster in MDCK and HeLa cells (see Supplementary Fig. S4C). All together, we demonstrated that transport of UPs across the GA triggers fragmentation of the Golgi ribbon in UCs as well as in UP-expressing MDCK and HeLa cells.

Fourth, since the GA undergoes irreversible fragmentation during apoptosis, in part as a result of caspase-mediated cleavage of several Golgi-associated proteins^38^, we next analyzed whether the synthesis of UPIb-EGFP and UPIb/UPIIIa-EGFP activate caspase 3, which plays a central role in the execution-phase of cell apoptosis. Confocal images did not reveal caspase 3 activation in UPIb-EGFP or UPIb/UPIIIa-EGFP expressing MDCK and HeLa cells (see Supplementary Fig. S5). The image analysis revealed that there was no active caspase 3 positive UP expressing UCs (n = 3) and MDCK cells (n = 4), and only 3 (6.7%) of UP expressing HeLa cells (n = 45) were also active caspase 3 positive. Altogether, only 5.8% of all analyzed UP expressing cells were active caspase 3 positive. Therefore it turns out that UPs cause fragmentation of the GA through their trafficking along the secretory pathway and not through induction of apoptosis.

### UPIb/UPIIIa-EGFP exit from the GA in COPI-, COPII- and clathrin-negative transport carriers

**Figure 6:**
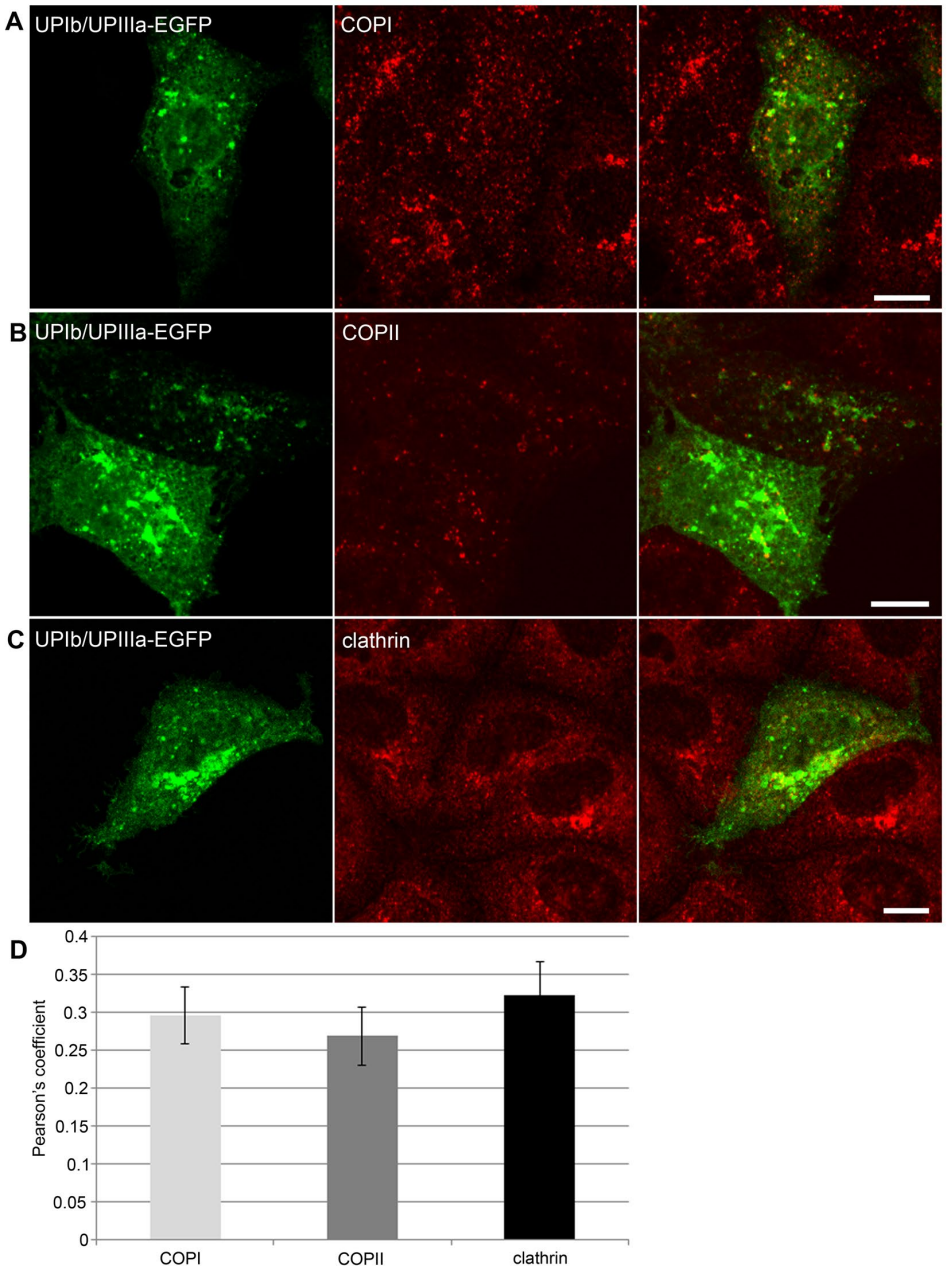
Distribution of UPIb/UPIIIa-EGFP signal and COPI, COPII or clathrin signal in MDCK cells. After synchronization of protein released from GA, MDCK cells were treated with tannic acid to prevent fusion of UPIb/UPIIIa-EGFP positive vesicles with the plasma membrane. (**A**–**C**) Representative images of the localization of UPIb/UPIIIa-EGFP (green) and other vesicle markers (red), as indicated. (**D**) Quantitative analyses of the localization of UPIb/UPIIIa-EGFP (green) and other vesicle markers show moderate co-localization of UPIb/UPIIIa-EGFP and COPI, COPII or clathrin. The degree of co-localization from the Pearson’s value correlation coefficients was categorized according to^69^ as very strong (0.85 to 1.0), strong (0.49 to 0.84), moderate (0.1 to 0.48), weak (−0.26 to 0.09), and very weak (−1 to −0.27). Error bars are the standard errors of the means (n = 3). Bars: 10 µm (**A–C**).

To check whether the peripheral UPIb/UPIIIa-EGFP vesicles exiting from the GA contain any of coated proteins, the immunofluorescence labeling of COPI, COPII and clathrin was performed on MDCK cells after synchronization of the protein release with brefeldin A and temperature block, and further prevention of the vesicle fusion with the PM by tannic acid treatment. The colocalization between UPIb/UPIIIa-EGFP and COPI, COPII or clathrin was analyzed in z-stacks acquired by the confocal microscope. Representative images show rare overlap of UPIb/UPIIIa-EGFP signals and signals of COPI (Fig. 6A), COPII (Fig. 6B) and clathrin (Fig. 6C). Furthermore, quantitative analyses confirmed only a moderate degree of co-localization (Fig. 6D).

To further check the presence of coated proteins at the surface of UP-positive vesicles in UCs, which express UPs endogenously, the immunofluorescence labeling on cryo semi-thin sections was performed. We found no overlapping signals of UPs with COPI, or clathrin in UCs. However, as expected, there were some overlapping signals between giantin and COPI as well as between giantin and clathrin (Supplementary Fig. S6).

These results correlated well with our previous findings that constitutive post-Golgi carriers do not contain any of COPI, COPII and clathrin positive vesicles^39^. Our observations may therefore suggest that UPs follow the constitutive post-Golgi route to the apical PM.

### Microtubules and actin filaments are required for UP trafficking to the plasma membrane

**Figure 7:**
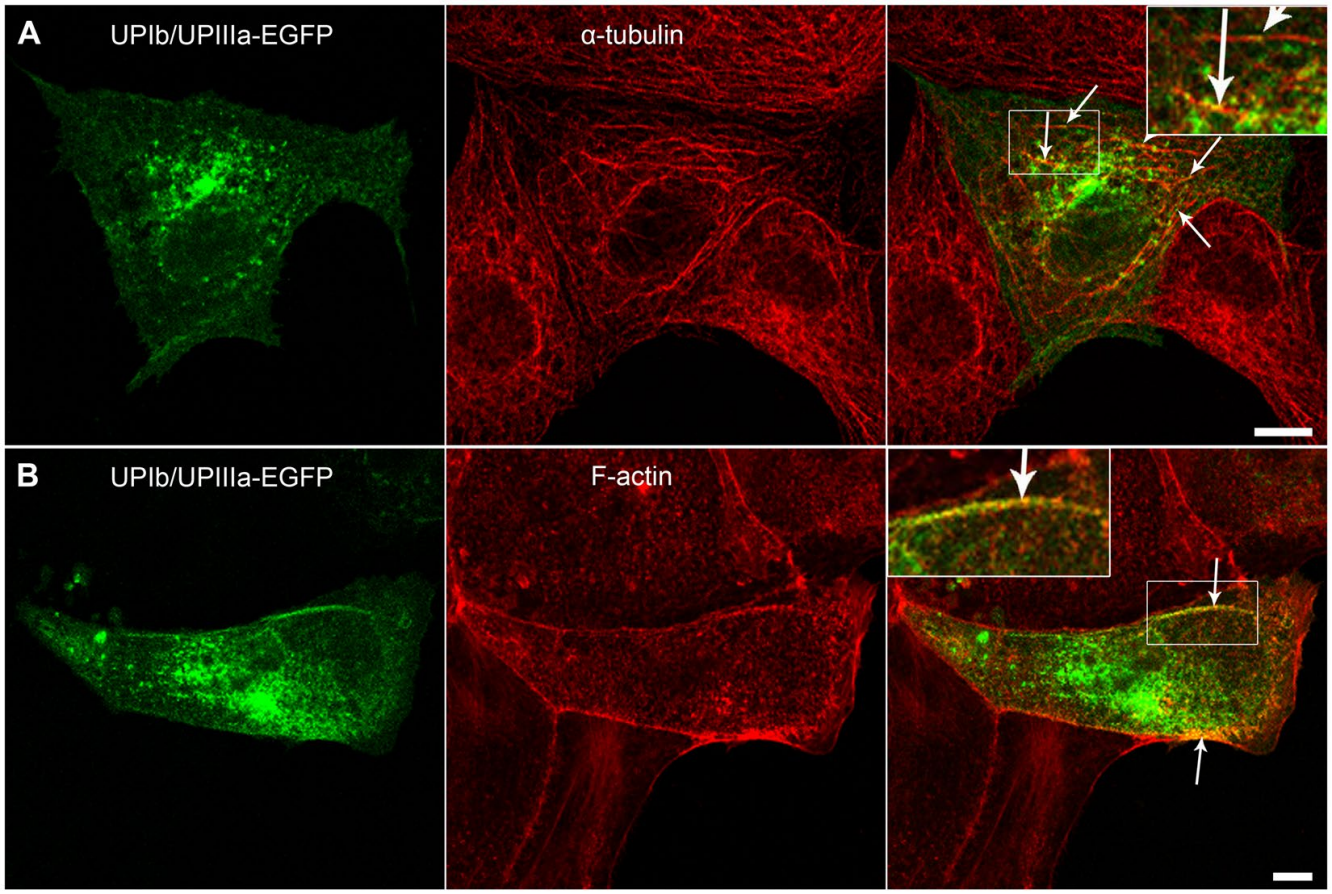
MTs and AFs mediate the transport of UPIb/UPIIIa-EGFP in MDCK cells. UPIb/UPIIIa-EGFP positive vesicles colocalize with (**A**) α-tubulin and (**B**) F-actin. (**A**) UPIb/UPIIIa-EGFP positive vesicles are seen on MTs (arrows). (**B**) UPIb/UPIIIa-EGFP positive vesicles near the PM co-localize with AFs (arrows). Areas in smaller frames are increased by 100% and presented in enlarged insets. Bars: 10 µm.

It was shown previously that rearrangement of MTs and AFs occurs during differentiation of UC^26,29,30^. Here we present evidence for the association of both MTs and AFs with UP-positive vesicles, which we assume to resemble discoidal or fusiform-shaped vesicles (DFVs) in UCs before reaching terminally differentiated stage. Immunofluorescence labelling showed a close association of UPIb/UPIIIa-EGFP containing vesicles with both α-tubulin and F-actin in MDCK cells (Fig. 7). UPIb/UPIIIa-EGFP vesicles overlapped with α-tubulin-positive microtubules throughout the cytoplasm (Fig. 7A) and with F-actin filaments only in the peripheral area of the cytoplasm underneath the PM (Fig. 7B).

**Figure 8:**
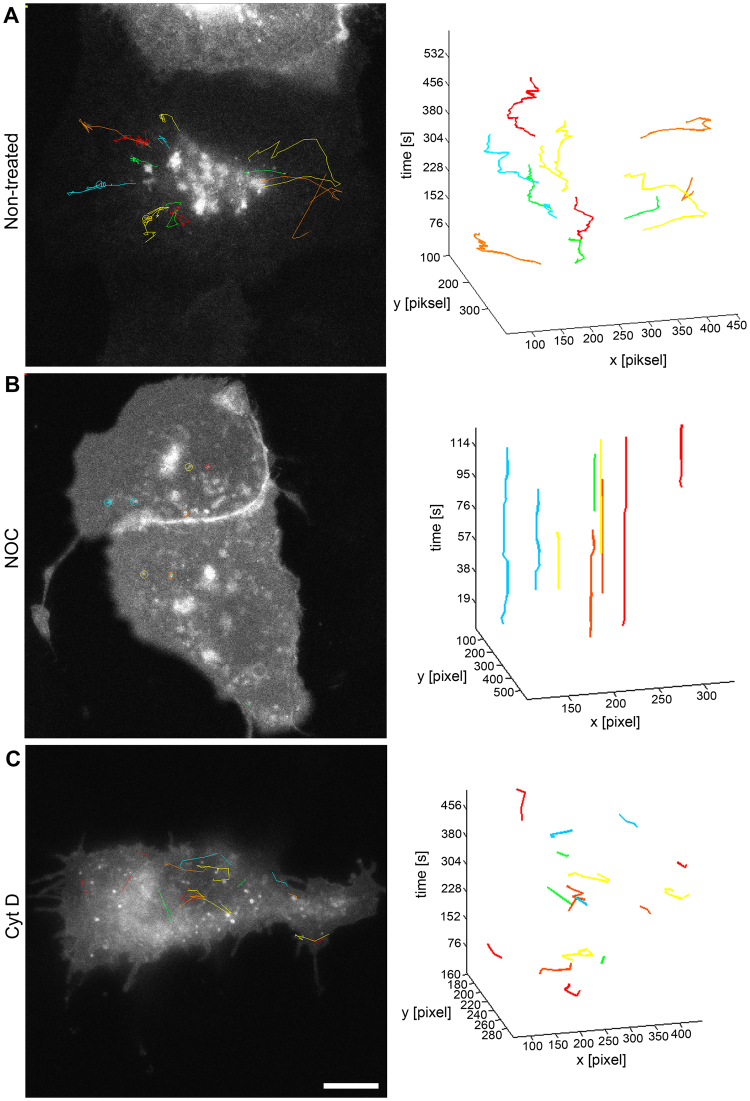
Dynamics of UPIb/UPIIIa-EGFP positive vesicles in non-treated, NOC and CytD treated MDCK cells. Movement of individual UPIb/UPIIIa-EGFP positive vesicles was monitored by time-lapse confocal microscopy in MDCK cells with synchronized protein release from the GA (**A**–**C**). Individual UPIb/UPIIIa-EGFP positive vesicles were tracked by software ParticleTR. The movement of UPIb/UPIIIa-EGFP positive vesicles and their trajectories are shown. (**A**) In non-treated UPIb/UPIIIa-EGFP expressing MDCK cells the UPIb/UPIIIa-EGFP transport proceeds normally from the GA to the PM. (**B**) The analysis shows that UPIb/UPIIIa-EGFP positive vesicles stop moving when the MTs are depolymerized. (**C**) The transport of UPIb/UPIIIa-EGFP is reduced, but not completely stopped when AFs are depolymerized. Bars: 10 µm (A-C).

We used time lapse confocal microscopy to analyze the dynamics of the transport from the GA to the PM by tracking individual UP positive vesicles (Fig. 8 and Supplementary Video S1). UPIb/UPIIIa-EGFP positive vesicles were not directly transported to the PM. Instead they moved in saltatory manner (transiently stopped or changed the direction). To evaluate the average velocity of UPIb/UPIIIa-EGFP positive vesicles we subtracted these halts and direction reversals. The average velocity of UPIb/UPIIIa-EGFP positive vesicles in UCs was 0.31 ± 0.01 µm/s (s.e.m.; n = 69) and in MDCK cells 0.35 ± 0.01 µm/s (s.e.m.; n = 98). The maximal velocity of UPIb/UPIIIa-EGFP positive vesicles in UCs was 1.71 µm/s and in MDCK cells 2.62 µm/s. That is consistent with the speed of the movement along microtubules^40^.

Next, we used time-lapse confocal microscopy to monitor the dynamics of UPIb/UPIIIa-EGFP transport in MDCK cells where the MTs were depolymerized with nocodazole (NOC). The movement of UPIb/UPIIIa-EGFP positive vesicles almost completely stopped indicating again the involvement of MTs in UPs transport (Fig. 8B and Supplementary Video S2).

To test the role of AFs in the transport of UPIb/UPIIIa-EGFP positive vesicles, we analyzed the dynamics of UPIb/UPIIIa-EGFP transport in MDCK cells incubated with cytochalasin D (CytD) to prevent polymerization of actin monomers. CytD-treated cells exhibited a significant decrease in the motility of UPIb/UPIIIa-EGFP positive vesicles and in their fusion with the PM (Fig. 8C and Supplementary Video S3).

In summary, the fluorescence immunolabelling of α-tubulin or F-actin with UPIb/UPIIIa-EGFP in MDCK cells, together with studies on the transport dynamics of UPIb/UPIIIa-EGFP positive vesicles in non-treated and NOC and CytD treated cells, provided the first evidence of the involvement of MTs and AFs in the transport of UPs to the PM.

## Discussion

Differentiation of the UCs includes many cellular mechanisms involved in the development of a specialized apical PM of superficial UCs that contains transmembrane proteins UPs. One of these cellular mechanisms requires the fragmentation of the GA^25,26^. Studies have suggested that the GA plays a central role in the complex formation of UPs, which are subsequently delivered to the apical PM during the differentiation of UCs^19,26^ and contribute to the formation of the tightest permeability barrier in the body^41,42^. However, the presence of UPs in the GA was never demonstrated *in situ*. It was shown that the GA undergoes structural rearrangement, which includes fragmentation of the GA and it’s spreading throughout the cytoplasm of the differentiating UCs^25,26^. The purpose of our study was therefore to elucidate the biosynthetic pathway of UPIb and UPIIIa from the ER to the PM with special emphasis on the question whether the presence and transport of UPs are involved in GA fragmentation.

In this study, we showed that UCs in the established cultures endogenously express all four UPs, Additionally, employing the FRIL technique, we confirmed the presence of UP particles in the apical PM of UCs. UP particles have been so far found only in highly differentiated superficial UCs (i.e. umbrella cells) of normal urothelium *in vivo* and in primary cultures of normal mouse UCs^24^.

With the transfection of established UC cultures with UPIb-EGFP or UPIb/UPIIIa-EGFP we ensured that EGFP-tagged UPs maintained ability to assemble with their partners and that they are delivered to the apical PM of polarized UCs. Our results showed that UPIIIa-EGFP can exit the ER in UCs, which endogenously express its partner UPIb, however it cannot exit the ER alone in 90% of the transfected MDCK cells (see Supplementary Table S3). Similar results were obtained also with 293 T cell^13^. We showed that UPIIIa-EGFP can be found in apical PM of MDCK cells when cotransfected with its partner UPIb. Therefore we conclude that the assembly of UPIIIa-EGFP with UPIb is necessary to be able to exit the ER.

UPIb-EGFP and UPIb/UPIIIa-EGFP have been found in the apical PM of polarized MDCK cells demonstrating that UPIb and UPIb/UPIIIa are directed to the apical PM also in cells which do not usually express UPs. Delivery of UPIb and UPIIIa to the apical PM suggests that at least UPIb contains the sorting signal that direct UPIb and UPIb/UPIIIa to the apical PM. Apical sorting signals can be localized to the cytoplasmic, luminal, and transmembrane domains of the proteins^43^. GPI-anchors, and N- and O-glycans attached to the membrane proteins can act as an apical sorting signal^44^. However, the sorting signals and sorting mechanisms of UPs remain to be discovered.

Newly synthesized proteins that leave the ER usually pass through the GA before being sorted for transport to their final destination. The glycosylation of UPIb, UPII, and UPIIIa as well as cleavage of UPII by furin suggest UP transport through the GA^14,17^. However, UPs have never been detected in the GA cisternae *in situ*. Possibly this happened due to the properties of utilized antibodies, which recognize only mature UPs. Another explanation for this lack of UPs in the GA is that UPs bypass GA, as it is known for protein-tyrosine phosphatase CD45, peripherin/rds and myeloproliferative leukemia virus oncogene^45–47^. Nevertheless, our results showed that UPs can be revealed both in ER and in the GA. This argues that UPs take conventional ER-to-Golgi-to-PM trafficking pathway.

The GA undergoes irreversible fragmentation during apoptosis, which is due in part to caspase-mediated cleavage of Golgi-associated proteins^48^; however this was not the case in UPs-expressing cells used in our study. Our results show that UPIb and UPIb/UPIIIa expression is associated with the fragmentation of the GA in UCs which express UPs endogenously, and in MDCK and HeLa cells, which do not express UPs endogenously. The results of the quantification analysis of the number of GA fragments in MDCK and in HeLa cells during the UP transport in this study coincide with the number of GA fragments in highly differentiated UCs^26^. These results additionally confirm a relationship between increased UP expression and fragmentation of GA.

We believe that the fragmentation of the GA during the differentiation process of the cells is not an uncommon event amongst different cell types. Comparable fragmentation and redistribution of GA has been observed also in MDCK cells that secrete human growth hormone^49^ and in differentiated muscle cells^50^. Probably similar structural changes of the GA occur in other cell types as an adaptation to elevate synthesis of different proteins^51^ and secretory demand of the cell that requires secretory Golgi outpost to be moved as close as possible to the sites of cargo delivery to the PM. Furthermore, synchronized reorientation of the GA along with the microtubule-organizing center is thought to facilitate polarized secretion, thereby providing membrane and secreted products directly to the nearest PM site such as the leading edge in migrating cells^52^ or in our case flattened apical surface of UCs. Our results together suggest that fragmentation and spreading of the GA in UCs is related to the (1) level of the UP synthesis, (2) secretory demand of the UCs and (3) shape and size of the UCs.

Analysis of Golgi-derived structures that carry UPs to the PM revealed neither COPI nor COPII nor clathrin at their surface. Presumably, COPI-dependent vesicles are too small for the transport of large cargo aggregates including proteinaceous membrane thickenings in UCs (reviewed in^51^). This suggests that UP-containing structures are likely to use post-Golgi trafficking mechanisms, which are utilized by number of constitutively secreted cargo proteins^39,53–55^. One of the common features in these mechanisms is movement of post-Golgi carriers along MTs^56,57^ and involvement of AFs in post-Golgi secretion^58^.

We demonstrated the co-localization of MTs and AFs with UPIb/UPIIIa-EGFP positive vesicles and determined the velocity of them. The maximum velocity of UPIb/UPIIIa-EGFP (2.62 µm/s) implies that the transport depends on MTs, since dyneins and kinesins enable the velocity of 0.5–4 µm/s^59,60^. However, myosins on AFs only enable a velocity of under 0.7 µm/s^61^ and the average velocity of UPIb/UPIIIa-EGFP (0.35 µm/s) suggests that the transport of UPIb/UPIIIa-EGFP depends also on AFs. As expected from the immunolabeling results, analysis of UPIb/UPIIIa-EGFP transport in cells with depolymerized MTs and AFs confirmed the involvement of MTs and AFs in UP transport. In the presence of the MT-disrupting agent NOC, UPIb/UPIIIa-EGFP positive vesicle movement almost completely stopped, failed to acquire directed motility, and remained stationary in GA. In contrast, inhibition of AF formation with CytD resulted in a slow motility with inability in the targeting of UPIb/UPIIIa-EGFP positive vesicles to the PM. Our results suggest that UPIb/UPIIIa-EGFP positive vesicles from GA to PM first move along MTs, which enable transport of higher velocity. However, closer to the PM UPIb/UPIIIa-EGFP positive vesicles passes to AFs, which enable lower velocity of transport, and therefore prepare UPIb/UPIIIa-EGFP positive vesicles for targeting to the PM.

We propose that upper described mechanism of UP-trafficking prevails in still differentiating UCs. When UCs become terminally differentiated with the bulk of UPs already at the apical PM, the MTs and AFs are reorganized^26^. MT organization in the basal, central and subapical cytoplasm of highly differentiated UCs is diminished and AFs are mainly depleted from the subapical cytoplasm^30,62^. In contrary, intermediate filaments are accumulated in the apical cytoplasm, which probably hinder MT-dependent centralization of GA and maintain the peripheral fragmentation of GA in differentiated UCs.

In summary, in this study new perspectives have emerged regarding the explanation of the UPs biosynthetic pathway and differentiation-dependent GA fragmentation. We showed that normal porcine UCs in culture can express all four UPs and are therefore a useful model for studies of dynamic processes that are comparable to normal UC of urothelium *in vivo*. Our results show that EGFP tagged UPs preserve their ability to form heterodimers and are correctly delivered to the PM. The passage of UPs (Ib, IIIa) through the GA induces its fragmentation not only in UCs, but also in cells that do not express UPs endogenously. MTs support the traffic of UP-positive vesicles from the GA while AFs contribute to their delivery to the PM. Although GA fragmentation was so far very well recognized in conjunction with mitosis^63^ and apoptosis^38^, we propose that GA fragmentation can also be needed for delivery of certain specific cargoes to PM. It will be important to understand how the trafficking machinery of the cells drives GA fragmentation in response to expression of such cargo proteins and whether such adaptation of the secretory system requires changes in the transcriptional program of the cells during differentiation.

## Methods

### Cell cultures

Cell cultures of normal UCs were established from normal porcine urinary bladders^26,32^. The experiments were approved by the Veterinary Administration of the Slovenian Ministry of Agriculture and Forestry in compliance with the Animal Health Protection Act and the Instructions for Granting Permits for Animal Experimentation for Scientific Purposes.

Urinary bladders were cut into 5 cm long and 2 cm wide strips and UCs were gently scraped from the urothelium with a scalpel and collected in UroM medium as described^32^. After collection of UC, the cells were centrifuged at 200 g for 5 min, washed in UroM medium and filtered through a 40 µm Cell strainer (BD Falcon^TM^, BD Biosciences, New Jersey, USA) to obtain a single-cell suspension. Primary and subsequent subcultures were plated with a seeding density of 2 × 10^5^ cells/cm^2^. They were grown in UroM with 0.9 mM calcium and 2.5% FBS (Gibco) until confluence and then transferred to UroM with 2.7 mM calcium and without FBS^64,65^. For the experiments in this study, UCs from the IV to X passages were used. When non-polarized UCs were acquired, the experiments were performed 24 h after the plating and when polarized UCs cells were acquired, the experiments were performed 5 days after the plating. To obtain highly differentiated UCs, the cells were cultured for 2 months.

MDCK cells were maintained in DMEM (Clonagen Inc, Gentaur, Belgium) supplemented with 10% FBS (EuroClone®, Pavia, Italy), 1 mM L-glutamine (Sigma‒Aldrich), 5% sodium pyruvate (Gibco), 5% non-essential amino acid (Gibco), 7.5% sodium hydrogen carbonate (Sigma‒Aldrich)_,_ and 1% Penicillin-Streptomycin (Sigma‒Aldrich). When non-polarized MDCK cells were acquired, the experiments were performed 24 h after the plating and when polarized MDCK cells were acquired, the experiments were performed 5 days after the plating.

HeLa cells were maintained in DMEM (Gibco) supplemented with 10% FBS (EuroClone®), 1 mM L-glutamine (Sigma‒Aldrich), and 1% Penicillin-Streptomycin (Sigma‒Aldrich). All experiments were performed 24 h after the plating.

All three cell types were cultured until use at 37 °C in a 95% humidified atmosphere with 5% CO_2_.

### RNA preparation and RT-qPCR

To determine if 2 months cultured UCs synthesize UPIb and UPIIIa the RT-qPCR was performed. UCs were homogenized with TRIzol reagent and lysed directly in the culture dish. Total RNAs from these cells were purified according to the manufacturer’s instructions. Total RNA (1 μg) was reverse-transcribed by QuantiTect Reverse Transcription kit (Qiagen) according to the manufacturer’s instructions.

RT-qPCR experiments were performed using Light Cycler 480 Syber Green MasterMix (Roche) for cDNA amplification and LightCycler 480 II (Roche) for signal detection. qPCR results were analyzed using the comparative Ct method normalized against housekeeping gene GAPDH. Primer sequences for RT-qPCR assays are shown in Table 1.

**Table 1:** Primer sequences for RT-qPCR assays.

Gene	Forward primer	Reverse primer
UPKIb	AGCCTCTACCCGCTGCTTGA	GGAAGAGGTTGGGTGTGAAA
UPKIIIa	TCGTTATCACGTCCATCCTG	CAGACGTGTATGAAGGCTCC
GADPH	TGCACCACCAACTGCTTGGCA	GGCATGGACCGAGGTCATGAG

### Freeze-fracture replica immunolabelling (FRIL)

For the detection of the intramembrane distribution of UPs, the FRIL technique was performed as described previously^24^. UPs were immunolabelled with a rabbit polyclonal antibody against all four UPs (anti-UPs)^1^. After washing in PBS, goat anti-rabbit IgG coupled to 18-nm gold particles were applied (Jackson Immunoresearch, West Grove, NA, USA). Immunolabelled replicas were observed in a Philips 410 transmission electron microscope (FEI, Eindhoven, The Netherlands).

### cDNA constructs of UPs

We generated four UP cDNA constructs. The UPIb and UPIIIa cDNA clones from DNASU Plasmid Repository were amplified by PCR using forward and reverse primers (see Supplementary Table S1). The PCR product was then ligated with a pcDNA3 vector (Clontech Laboratories, Inc., Takara Bio Europe AB, Göteborg, Sweden) or pEGFP-N1 (Invitrogen^TM^) using restriction sites inserted in the forward and reverse primers (Sigma‒Aldrich). All DNA constructs were sent to DNA sequencing (Primm, Italy). The glycosylphosphatidylinositol (GPI-GFP) construct was kindly provided by Prof. Jennifer Lippincott-Schwartz (NIH, Bethesda, MD).

### Cell transfection

According to the manufacturer’s instructions, UCs, MDCK, and HeLa cells were transfected with NanoJuice (Merck, Darmstadt, Germany), Lipofectamin 2000 (Invitrogen) or Mirus (Mirus Bio LLC, Wisconsin, USA), respectively. The transfection was performed 24 h after plating the cells. Combinations of used constructs in different cell-types are presented in Supplementary Table S2. Transfection with GPI-GFP was performed as a control, which presented cells with increased non-relevant membrane protein. After transfection, the cells were washed with a cell-type specific medium and incubated overnight. When non-polarized MDCK or HeLa cells were used, the further experiments were performed 24 h after the transfection. When polarized UCs or MDCK cells were used, the further experiments were performed 5 days after the transfection.

### Fluorescence labelling

The cells were fixed in 4% formaldehyde for 10 min at room temperature, washed in PBS and preincubated with blocking buffer (0.5% BSA, 0.1% saponin, and 50 mM ammonium chloride in PBS). Immunolabelling was done with the following mouse monoclonal antibodies: anti-ZO-1 (1:20; Zymed Laboratories, Thermo Fisher Scientific, Waltham, MA, USA), anti-giantin (1:1000; Enzo Life Sciences, Farmingdale, New Yourk, USA), anti-GM130 (1:500; BD Transduction Laboratories, BD Biosciences, New Jersey, USA), anti-α-tubulin (1:100; Sigma‒Aldrich) anti-golgin 97 (1:1000; BD Transduction Laboratories), anti-occludin (1:50; BD Transduction Laboratories), anti-COPI (1:800; BD Transduction Laboratories), anti-COPII (1:100; BD Transduction Laboratories), anti-clathrin (1:1000; Abcam, Cambridge, UK); and polyclonal rabbit antibodies: anti-UPIb (1:200), anti-UPIIIa (1:50) and anti-UPs (1:1000); all three were a gift from Prof. Tung-Tien Sun, New York University, School of Medicine), anti-GFP (1:250; Abcam), and anti-caspase 3 (1:1000; R&D Systems, Minneapolis, USA). After washing in PBS, goat anti-rabbit and goat anti-mouse IgG conjugated with Alexa Fluor 488 or Alexa Fluor 555 were applied (1:400; Molecular Probes®, Invitrogen^TM^, Life technologies, Thermo Fisher Scientific, Waltham, MA, USA).

For labelling of F-actin, the cells were fixed in 4% formaldehyde for 10 min. After fixation, cells were washed in PBS and labelled with TRITC-labelled phalloidin (1:5, Sigma‒Aldrich) for 30 min at room temperature in the dark.

For ER labelling the ER-Tracker^TM^ Blue-White DPX (Life Technologies) was used according to the manufacturer’s instructions. Live transfected cells were stained with 1 µM ER-Tracker for 20 min at 37 °C. The staining solution was removed and the cells were washed with a cell-type specific medium and fixed in 4% formaldehyde for 10 min. After labeling the cells were washed in PBS, mounted with Mowiol (Sigma‒Aldrich) or with DAPI-Vectashield (Vector Laboratories, Cambridgeshire, UK) and then examined with an inverted confocal microscope Zeiss LSM710 (Carl Zeiss, Gottingen, Germany) or with a fluorescence microscope AxioImager.Z1 (Carl Zeiss MicroImaging GmbH) with an ApoTome attachment for optical sectioning and three-dimensional reconstruction.

### Immunofluorescence labeling of cryo semi-thin sections

Two-month urothelial models of normal porcine UCs were fixed in 4% formaldehyde in 0.1 M phosphate buffer, rinsed in PBS/20 mM Glycine, gently scraped from the surface, embedded in 12% gelatin, infused with 2.3 M sucrose and frozen in liquid nitrogen. Sections (300 nm thick) were cut at −80 °C, washed with PBS/20 mM Glycine and blocked with 1% BSA. Single immunolabeling of uroplakins was performed with rabbit polyclonal antibodies against UPIa (1:500), UPIb (1:500), UPII (1:100) and mouse monoclonal antibody against UPIIIa (a gift from Prof. Tung-Tien Sun, New York University, School of Medicine). Appropriate IgGs-AlexaFluor488 were used as secondary antibodies (1:400). For double immunolabeling the following combinations of antibodies were used: (i) mouse anti-COPI (1:1500; ref.^66^) and rabbit anti-UPs (1:10000; a gift from Prof. Tung-Tien Sun, New York University, School of Medicine), (ii) mouse anti-COPI and rabbit anti-giantin (1:1000; provided by Prof. Antonella De Matteis, TIGEM, Napoli), (iii) mouse anti-clathrin (1:300; BD Transduction Laboratories) and anti-UPs, (iv) mouse anti-clathrin and anti-giantin. Goat anti-mouse IgG with AlexaFluor488 and goat anti-rabbit IgG with AlexaFluor555 (both 1:400, Thermo Fisher Scientific) were used as secondary antibodies.

### Scanning electron microscopy

Two-month urothelial models of normal porcine UCs were fixed in 4% (w/v) formaldehyde and 2.5% (v/v) glutaraldehyde in 0.1 M cacodylate buffer, pH 7.4 for 2 h 45 min at 4 °C. The samples were rinsed in 0.1 M cacodylate buffer and postfixed in 1% osmium tetroxide in the same buffer for 1 h at 4 °C. Specimens were critical-point dried, sputter-coated with gold, and examined at 15 kV with a Jeol JSM 840 A scanning EM.

### Immunoelectron microscopy

The MDCK and HeLa cells transfected with UPIb-EGFP or UPIb/UPIIIa were fixed as described previously^66,67^. Cells were incubated with the anti-EGFP antibody (1:250; Abcam), washed in PBS and incubated in anti-rabbit Fab’ fragments coupled to 1.4-nm nanogold (1:100; Molecular Probes). Nanogold was enlarged by the GoldEnhance protocol (Nanoprobes Inc., Yaphank, NY, USA). Sections of 65-nm thickness were collected and EM images were acquired by a Techai^TM^-G^2^ Spirit electron microscope equipped with a VELETTA CCD digital camera (FEI, Eindhoven, The Netherlands).

### Quantitative analysis of GA structure

To determine the influence of the UP transport on GA structure, the MDCK and HeLa were first transfected with specific cDNA constructs (see Supplementary Tables S1 and S2) and then immunolabelled with anti-giantin and anti-UP antibodies. Ten random 3D images were taken with a confocal microscope Zeiss LSM710 (Carl Zeiss, Gottingen, Germany) and then the optical sections were analyzed (*z*-axis step set to 0.365 μm) as described previously^26^. Briefly, the number of GA fragments visible in Z-stacking panels of the GA in UP-expressing cells was counted with ParticleCO software (Celica, Ljubljana, Slovenia). With the custom software tool based on Matlab (Matworks, Natick, USA), we computed the average Euclidean distance between all pairs of GA fragments in each cell. Then we classified all fragments into clusters by creating a hierarchical cluster tree from Euclidean distances. The criterion for characterization was the inconsistency coefficient, which was set to a value of one. To find the variability in the number of clusters and in the number of fragments, the number of fragments per cluster was calculated for each cell. Data were analyzed using an analysis of variance (ANOVA). Differences with a *p* value < 0.05 were considered statistically significant.

### The synchronization of protein release from GA with brefeldin A

To examine the dynamics of the UPIb/UPIIIa- EGFP-transport, the synchronization of protein release from GA was induced. Transfected MDCK cells were incubated overnight with 5 µg/ml brefeldin A (Sigma-Aldrich) that blocks movement of newly synthesized membrane proteins from the ER to the GA^68^. Cells were then washed with culture medium with 0.02 M Hepes and shifted from 37 °C to 20 °C for 2 h to allow accumulation of UPs in the GA. After this temperature block, cells were shifted to 37 °C to monitor UP release from the GA. Samples were either analyzed with time-lapse confocal microscopy or prepared for immunofluorescence and immunoelectron microscopy. Synchronization of protein release from the GA in UCs and HeLa cells was not achieved, since UCs did not survive the treatment with brefeldin A and HeLa cells were not responding to treatment.

### Tannic acid treatment for prevention of the vesicle fusion with the PM

To study the correlation between the UPIb/UPIIIa-EGFP signals and COPI, COPII and clathrin signals, first the synchronization of protein release from GA was induced with brefeldin A. After synchronization of proteins, the MDCK cells were washed with culture medium with 0.02 M Hepes. Then the cells were shifted from 37 °C to 20 °C for 2 h to accumulate proteins in the GA. After this temperature block, cells were shifted to 32 °C for 45 min and treated with 0.5% tannic acid (Fluka, Sigma‒Aldrich) to prevent fusion of UPIb/UPIIIa-EGFP positive vesicles with the plasma membrane. Samples were then prepared for immunofluorescence microscopy using antibodies against COPI, COPII and clathrin proteins.

### Quantification of co-localization

For the quantification of co-localization, the Pearson’s correlation coefficients were calculated from the raw optical sections for the pairing of UPIb/UPIIIa-EGFP and the other vesicle markers (COPI, COPII, clathrin). The Pearson’s correlation coefficients were then calculated using the JaCoP plugin (Image J) from three z-stack images. The Pearson’s coefficients were averaged, and a standard error of the mean was calculated. The degree of co- localization from the Pearson’s coefficients was categorized according to^69^: very strong (0.85 to 1.0), strong (0.49 to 0.84), moderate (0.1 to 0.48), weak (−0.26 to 0.09) and very weak (−1 to −0.27).

### Nocodazole and cytochalasin D treatment

To study the influence of MTs and AFs on the UP transport we synchronized protein release from the GA in transfected MDCK cells by brefeldin A, as described above. During UP accumulation in the GA at 20 °C, the cells were treated with 66 μM nocodazole (NOC) for 15 min or 20 µg/ml cytochalasin D (CytD) for 30 min to disassemble MTs and AFs, respectively. The cells were then transferred to 37 °C to perform time lapse confocal microscopy.

### Time lapse confocal microscopy

Dynamics of UP transport in nocodazole and cytochalasin D treated MDCK cells was analyzed by time lapse confocal microscopy. The details of the method are described in ref.^70^. Briefly, the time-lapse images of EGFP-labeled UPs in cells were acquired every 3.8 s with an inverted confocal microscope Zeiss LSM710 (Carl Zeiss, Gottingen, Germany) with a microscope stage incubator chamber that allows controlled conditions (37 °C, 95% humidity, 5% CO_2_). The EGFP was excited with the 488 nm line of a krypton-argon laser and an appropriate band filter was used. The time-lapse images were acquired with the ZEN Time Lapse module (Carl Zeiss, Gottingen, Germany) and analyzed by ParticleTR software (Celica, Ljubljana, Slovenia; http://celicabiomedical.com/Docs/ParticleTR.pdf). To avoid experimental error due to cell movement, only cells that did not move relative to the substratum during the acquisition were analyzed. The instantaneous rate of transport was determined by dividing the distance travelled by a vesicle with time, excluding the time when the vesicle halted or reversed its direction.

## Electronic supplementary material

Supplementary Video S1. Dynamics of UPIb/UPIIIa-EGFP positive vesicles in MDCK cells.

Supplementary Video S2. Dynamics of UPIb/UPIIIa-EGFP in MDCK cells where the MTs were depolymerized with nocodazole.

Supplementary Video S3. Dynamics of UPIb/UPIIIa-EGFP in MDCK cells where the AFs were depolymerized with cytochalasin D.

Supplementary information 
