# Supplementary material for: Uroplakin traffic through the Golgi apparatus induces its fragmentation: new insights from novel in vitro models
Source: Sci Rep. 2017 Oct 9;7:12842. doi: 10.1038/s41598-017-13103-x (PMC5634464; doi:10.1038/s41598-017-13103-x)
Supplement: Supplementary file 4 — Supplementary information [file 41598_2017_13103_MOESM4_ESM.pdf]

SUPPLEMENTARY INFORMATION

Uroplakin traffic through the Golgi apparatus induces its fragmentation: new insights from novel in vitro models

[1]Tanja Višnjar, [2]Giancarlo Chesi, [2]Simona Iacobacci, [2]Elena Polishchuk, [1]Nataša Resnik, [3]Horst Robenek, [4]Marko Kreft, [1]Rok Romih, [2]Roman Polishchuk*, [1]Mateja Erdani Kreft*

[1]Institute of Cell Biology, Faculty of Medicine, University of Ljubljana, Vrazov trg 2, SI-1000 Ljubljana, Slovenia
[2]Telethon Institute of Genetics and Medicine (TIGEM), Via Campi Flegrei 34, 80078 Pozzuoli (NA), Italy
[3]Institute for experimental musculoskeletal medicine, University of Münster, Albert-Schweitzer-Campus 1, Domagkstrasse 3, 48149 Münster, Germany
[4]Department of Biology, Biotechnical Faculty, University of Ljubljana, Večna pot 111, Ljubljana, Slovenia & LN-MCP, Institute of Pathophysiology, Faculty of Medicine, University of Ljubljana & Celica Biomedical Center, Ljubljana, Slovenia

*Corresponding authors:
Kreft ME, Institute of Cell Biology, Faculty of Medicine, University of Ljubljana, Vrazov trg 2, SI-1000 Ljubljana, Slovenia

E-mail: mateja.erdani@mf.uni-lj.si

Tel.: +386 1 543 7685

Fax: +386 1 543 7681

Polishchuk R, Telethon Institute of Genetics and Medicine (TIGEM), Via Campi Flegrei 34, 80078 Pozzuoli (NA), Italy

E-mail: polish@tigem.it

Tel.: +39 081 6132336

Fax: +39 081 5609877

Supplementary Figures:

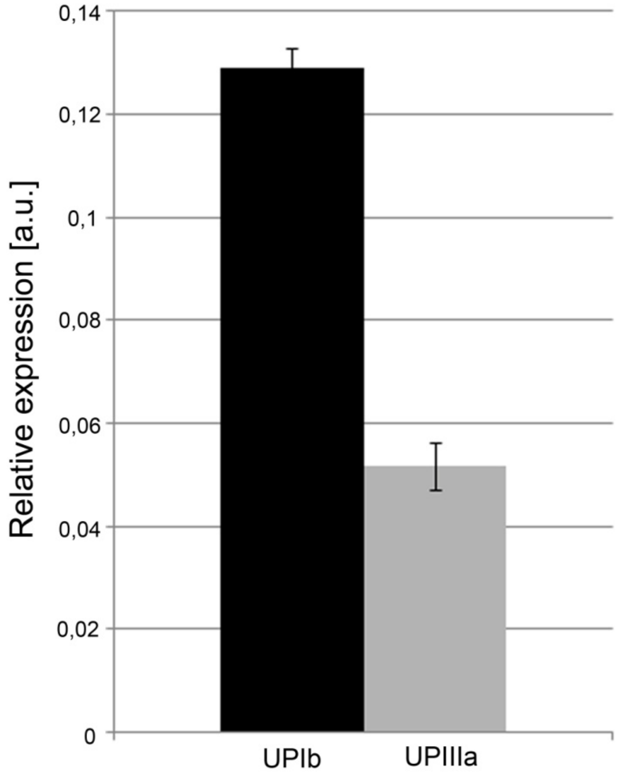

Supplementary Figure S1. UPIb and UPIIIa mRNA levels in UCs *in vitro*. Expression of UPIb and UPIIIa mRNA was analyzed by RT-qPCR assay in highly differentiated UCs cultured for 2 months. Expression values were calculated applying the $-2^{\Delta\Delta CT}$ algorithm. Estimate relative quantities were normalized for the expression value of GAPDH. Data are the mean [a.u.]. Error bars are the standard errors of the means (n=3).

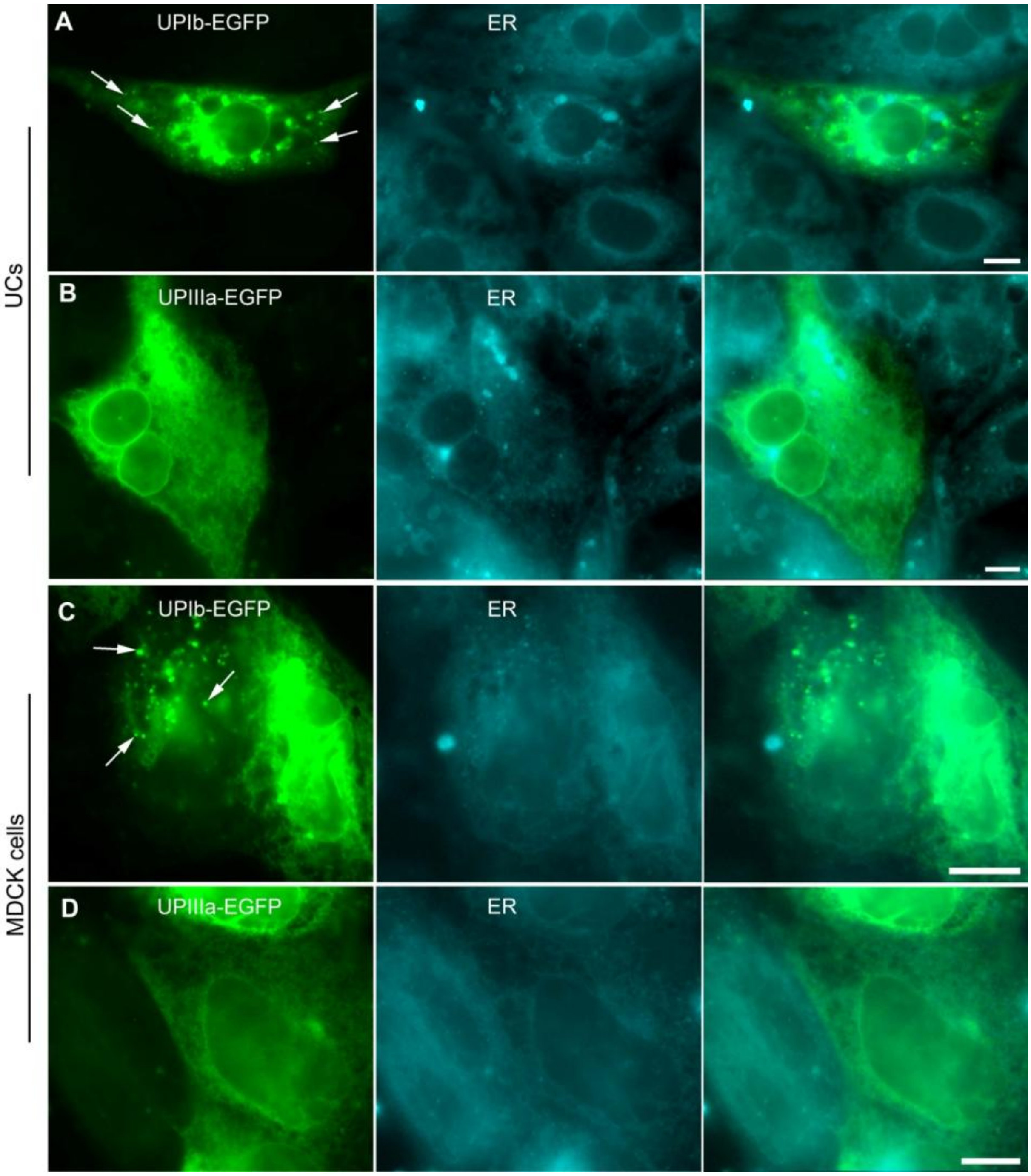

Supplementary Figure S2. Expression and distribution of UPIb-EGFP and UPIIIa-EGFP in non-polarized UCs and MDCK cells with labeled ER by ER-Tracker^{TM}. (A) UPIb-EGFP was found in ER and also outside the ER in non-polarized UCs (A, arrows) and MDCK cells (C, arrows). UPIIIa-EGFP was found only in ER of non-polarized UCs (B) and MDCK cells (D). Bars: 10 µm (A-D).

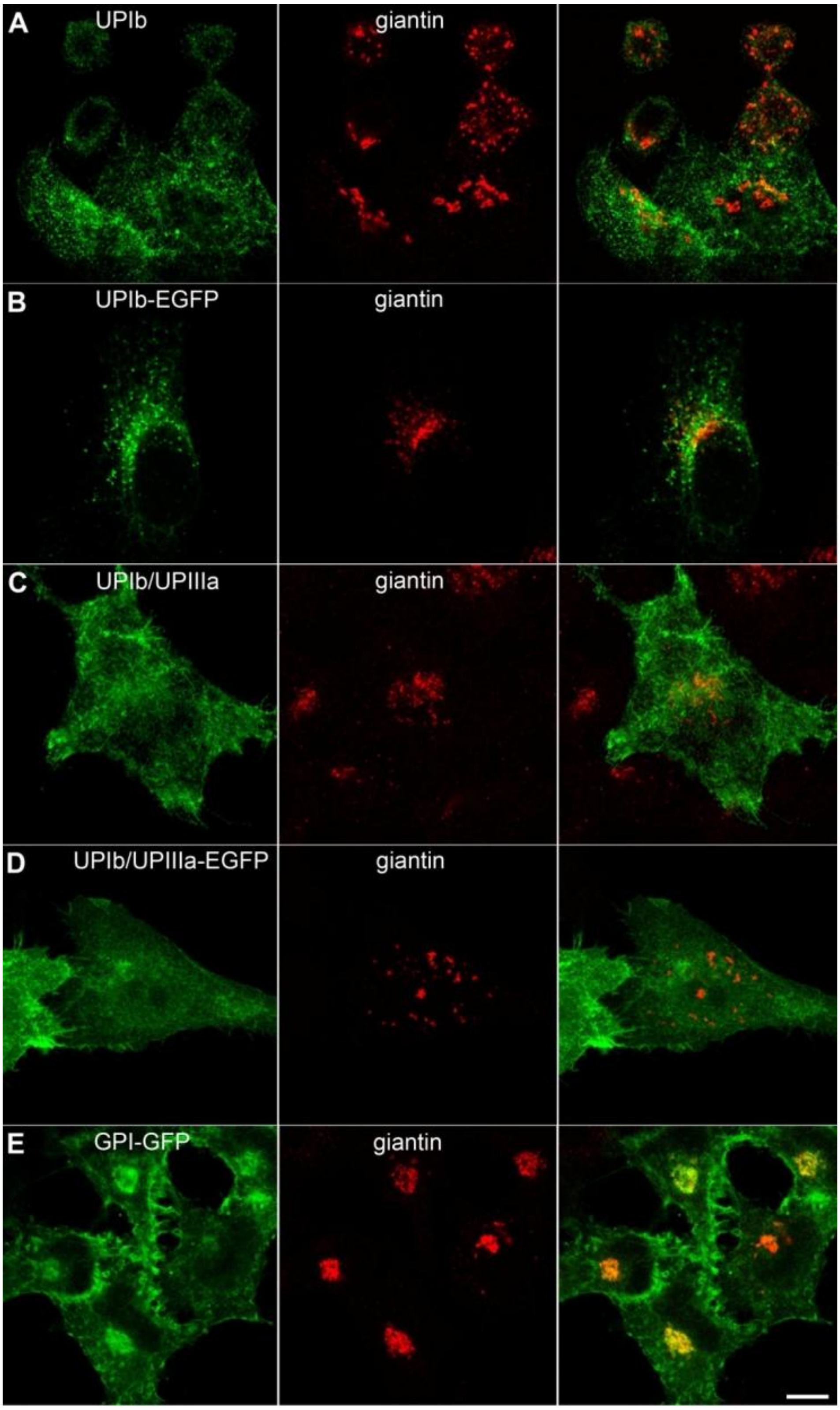

Supplementary Figure S3. GA structure in non-polarized HeLa cells transfected with (A) UPIb; (B) UPIb-EGFP, (C) UPIb/UPIIIa, (D) UPIb/UPIIIa-EGFP, or (E) GPI–GFP. Cells transfected with UPIb were immunolabelled with antibody against UPIb and cells transfected with UPIb/UPIIIa were immunolabelled with antibody against UPIIIa. (A, B) Anti-giantin immunolabeling shows that transport of untagged or EGFP-tagged UPIb causes

fragmentation of GA. (C, D) The transport of assembled heterodimers UPIb/UPIIIa or UPIb/UPIIIa-EGFP also causes fragmentation of GA. Note untagged and tagged heterodimers UPIb/UPIIIa on PM of HeLa cells. (E) The GA structure is not changed in GPI-GFP-expressing HeLa cells. Bars: 10 µm (A-E).

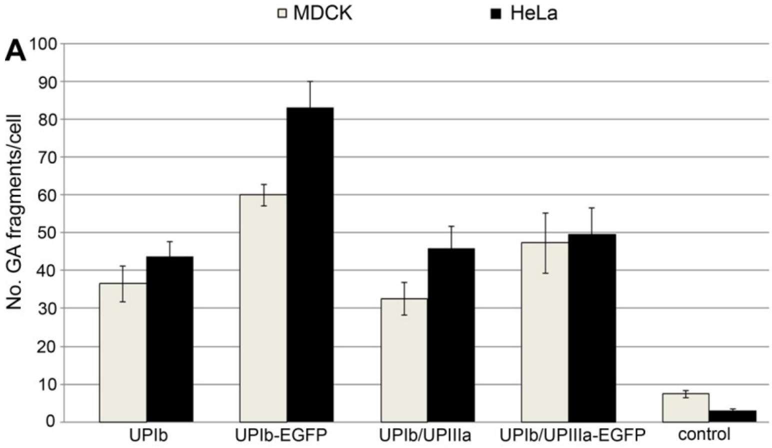

Statistical differences (ANOVA; p<0.05)		
MDCK	HeLa	No. of GA fragments/cell
no	yes	UPIb vs UPIb-EGFP
no	no	UPIb vs UPIb/UPIIIa
no	no	UPIb vs UPIb/UPIIIa-EGFP
yes	yes	UPIb vs control
no	yes	UPIb-EGFP vs UPIb/UPIIIa
no	yes	UPIb-EGFP vs UPIb/UPIIIa-EGFP
yes	yes	UPIb-EGFP vs control
no	no	UPIb/UPIIIa vs UPIb/UPIIIa-EGFP
yes	yes	UPIb/UPIIIa vs control
yes	yes	UPIb/UPIIIa-GEFP vs control

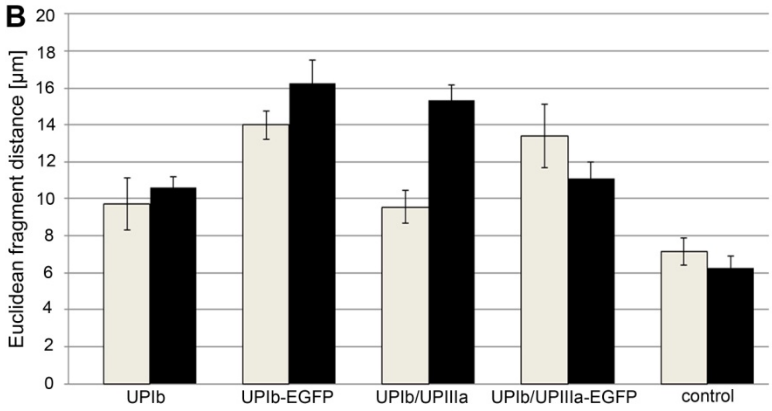

Statistical differences (ANOVA; p<0.05)		
MDCK	HeLa	Euclidean fragment distance
no	yes	UPIb vs UPIb-EGFP
no	yes	UPIb vs UPIb/UPIIIa
no	no	UPIb vs UPIb/UPIIIa-EGFP
no	yes	UPIb vs control
no	yes	UPIb-EGFP vs UPIb/UPIIIa
no	yes	UPIb-EGFP vs UPIb/UPIIIa-EGFP
yes	yes	UPIb-EGFP vs control
no	yes	UPIb/UPIIIa vs UPIb/UPIIIa-EGFP
no	yes	UPIb/UPIIIa vs control
yes	yes	UPIb/UPIIIa-GEFP vs control

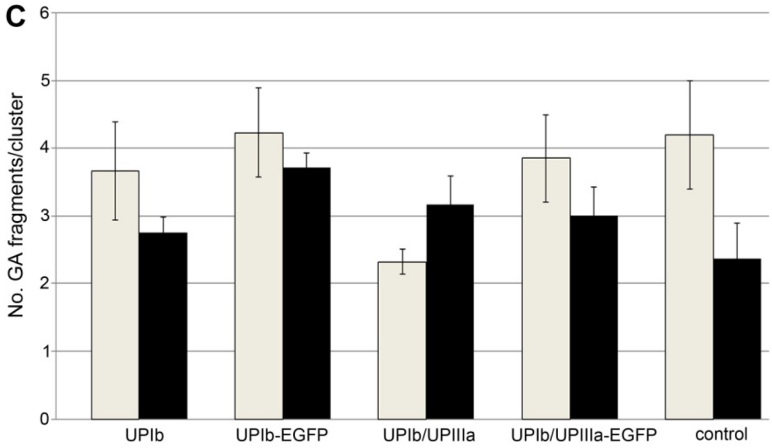

Statistical differences (ANOVA; p<0.05)		
MDCK	HeLa	No. of GA fragments/cluster
no	no	UPIb vs UPIb-EGFP
no	no	UPIb vs UPIb/UPIIIa
no	no	UPIb vs UPIb/UPIIIa-EGFP
no	no	UPIb vs control
no	no	UPIb-EGFP vs UPIb/UPIIIa
no	no	UPIb-EGFP vs UPIb/UPIIIa-EGFP
no	no	UPIb-EGFP vs control
no	no	UPIb/UPIIIa vs UPIb/UPIIIa-EGFP
no	no	UPIb/UPIIIa vs control
no	no	UPIb/UPIIIa-GEFP vs control

Supplementary Figure S4. Quantitative analysis of GA fragmentation shows statistical differences between UP-expressing and control MDCK and HeLa cells. To determine

changes of GA in UPs-expressing MDCK and HeLa cells, the difference in number of GA fragments per cell, Euclidean fragment distance and the number of GA fragments per cluster were analyzed using an analysis of variance (ANOVA; Bonferroni's multiple comparison test). Differences with a p value <0.05 were considered statistically significant (yes). (A) The average number of GA fragments (± s.e.m.) in transfected MDCK and HeLa cells is presented. We found significant increase in number of GA fragments per cell in all UP-expressing MDCK and HeLa cells in comparison to the control cells (written in red). (B) The Euclidean fragment distance is significantly increased in all UP-expressing cells, except in UPIb- and UPIb/UPIIIa- expressing MDCK cells, in comparison to the control cells. (C) Note that there is no significant difference in the number of GA fragments per cluster between the UP-expressing and control MDCK and HeLa cells. In average, the number of GA fragments per cluster in UP-expressing MDCK and HeLa cells is 3.

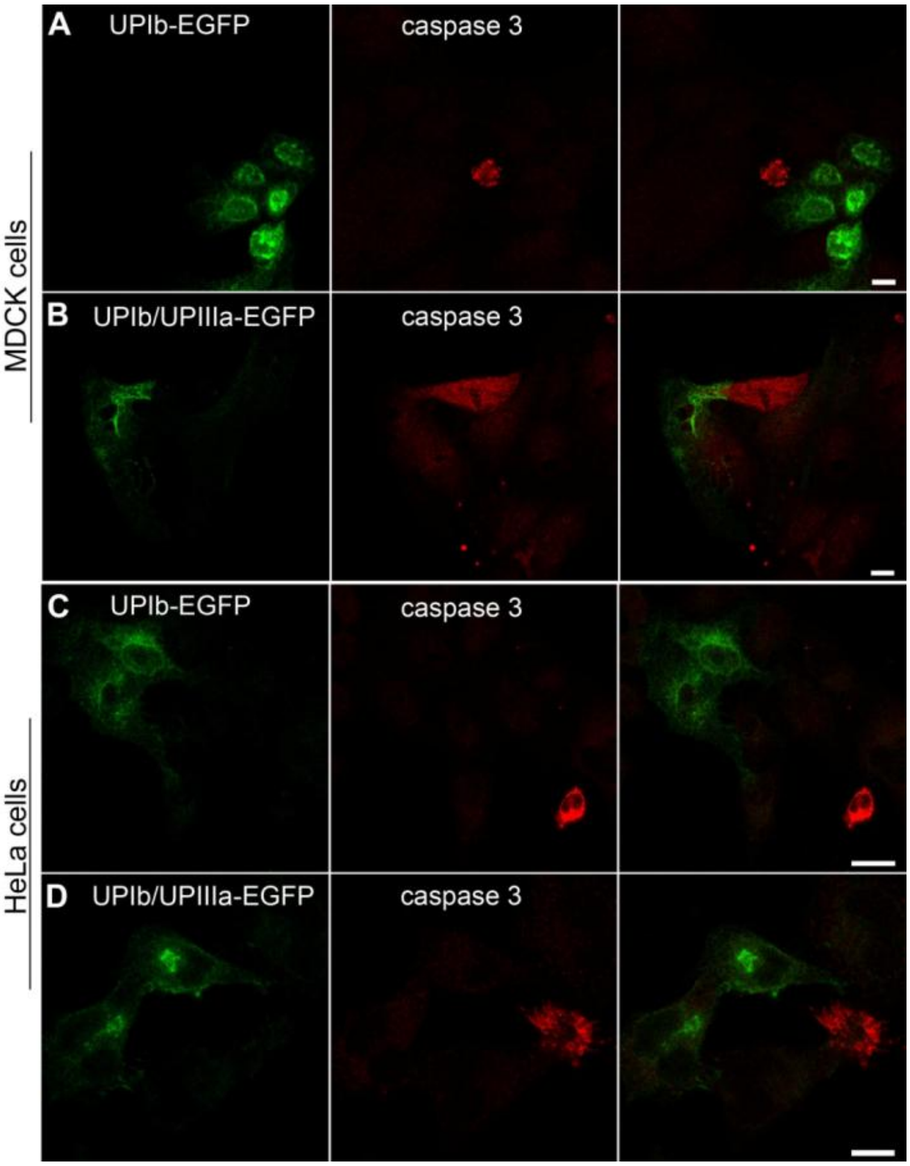

Supplementary Figure S5. Expression of UPIb-EGFP or UPIb/UPIIIa-EGFP in MDCK and HeLa cells do not trigger apoptosis. (A-D) The immunolabelling of active caspase 3 does not correlate with the expression of UPs. Cells with active caspase 3 do not express UPs. Bars: 20 µm (A) in 10 µm (B–D).

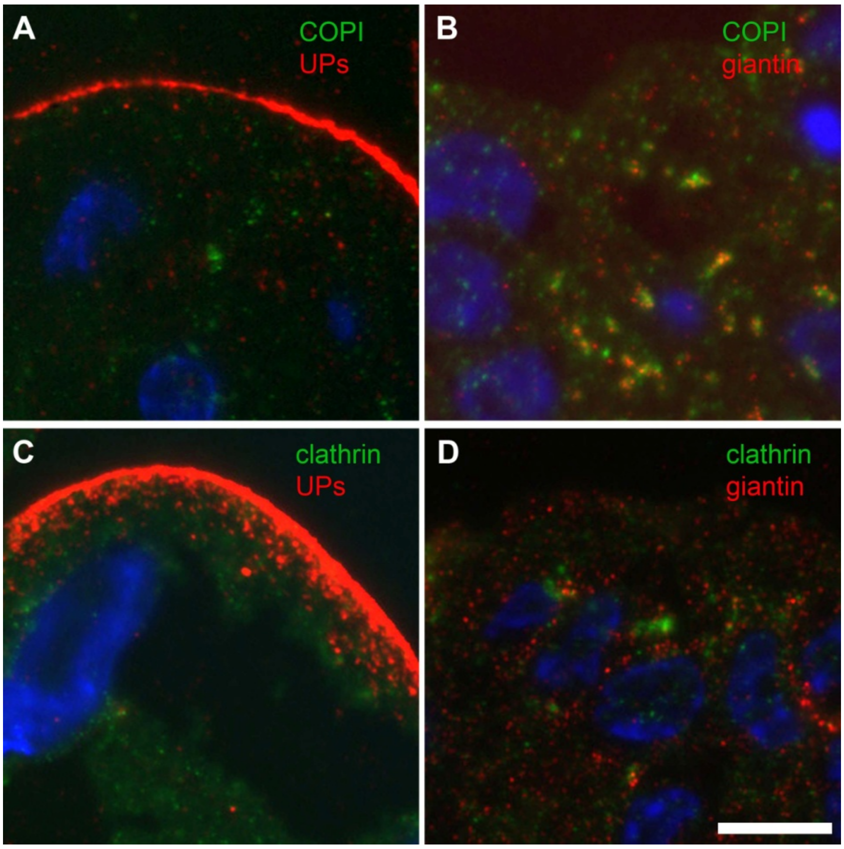

Supplementary Figure S6. Distribution of COPI, clathrin, UPs and giantin signals in cryo semi-thin sections of highly differentiated UCs cultured for 2 months. (A) The signal of COPI does not overlap with the signal of UPs. (B) Signals of COPI partially overlap with the signals of giantin. (C) Immunofluorescence labeling of (C) clathrin and UPs shows no overlapping signals, and (D) immunofluorescence labeling of clathrin and giantin shows rare overlapping signals. Nuclei (DNA) are labelled with DAPI (blue). Bar: 10 µm.

Supplementary Tables:

Name	**UPIb**	**UPIb–EGFP**	**UPIIIa**	**UPIIIa–EGFP**
Clone	HsCD00302119 (DNASU)	HsCD00302119 (DNASU)	HsCD00301184 (DNASU)	HsCD00301184 (DNASU)
Forward primer	CGCGGATCCACCATGGCCAAAGACAACTCA	CGCGAATTCTATGGCCAAAGACAACTCAACT	TTTGGTACCACGATGCCTCCGCTCTGGGC	TTTGAATTCTATGCCTCCGCTCTGGGCC
Reverse primer	CGCGAATTCTTAATATTCAATTCTGCTCCAGT	CTCGGATCCCAATATTCAATTCTGCTCCAGTAG	CTGGAATTCTCAGTCCTGGAGCTTGCTGG	ATTGGTACCCCGTCCTGGAGCTTGCTGGAAT
Restriction enzyme	BamHI and EcoRI	EcoRI and BamHI	KpnI and EcoRI	EcoRI and KpnI
Vector	pcDNA3.0	pEGFP-N1	pcDNA3.0	pEGFP-N1

Supplementary Table S1. cDNA constructs of UPs.

	UCs	MDCK cells	HeLa cells
UPIb		●	●
UPIb-EGFP	●	●	●
UPIb/UPIIIa		●	●
UPIb/UPIIIa-EGFP	●	●	●
UPIIIa-EGFP	●	●	
GPI-GFP		●	●

Supplementary Table S2. **Combinations of cDNA constructs used for transfection of UCs, MDCK and HeLa cells.** Bullets represent performed transfections in different cell types.

Localization of UPs in the cell	RIBBON GA [% of cells]	FRAGMENTED GA [% of cells]
UPIb-EGFP in ER	68	6
UPIb-EGFP in vesicles and PM	1	25
UPIIIa-EGFP in ER	83	7
UPIIIa-EGFP in vesicles	0	10
UPIb/UPIIIa-EGFP in ER	1	3
UPIb/UPIIIa-EGFP in vesicles and PM	29	67

Supplementary Table S3. Quantitative analysis of GA structure in non-polarized MDCK cells transfected with UPIb-EGFP, UPIIIa-EGFP or UPIb/UPIIIa-EGFP (n=234). Twenty-four hours after transfection with specific cDNA constructs, the immunolabeling with anti-giantin was performed. In all MDCK cells, which expressed UPIb-EGFP, UPIIIa-EGFP or UPIb/UPIIIa-EGFP the location of UPs and the form of GA was determined. Anti-giantin immunolabeling shows that fragmentation of GA increases when UPIb-EGFP, UPIIIa-EGFP or UPIb/UPIIIa-EGFP are transported through the GA to the PM.

Supplementary Videos:

Supplementary Video S1. Dynamics of UPIb/UPIIIa-EGFP positive vesicles in MDCK cells.

Time-lapse movie of MDCK cells expressing UPIb/UPIIIa-EGFP. Dynamics of UPIb/UPIIIa-EGFP positive vesicles (green) were analyzed by time-lapse confocal microscopy using a laser-scanning confocal microscope (LSM710, Carl Zeiss, Ins.). Frames were taken every 3.8 s for 10 min. Display rate is 15 frames/sec. Representative images are shown in Fig. 8A.

Supplementary Video S2. Dynamics of UPIb/UPIIIa-EGFP in MDCK cells where the MTs were depolymerized with nocodazole. Time-lapse movie of MDCK cells with depolarized MTs and expressing UPIb/UPIIIa-EGFP (green). Time-lapse confocal images were taken every 3.8 s for 2 min using a laser-scanning confocal microscope (LSM710; Carl Zeiss). Display rate is 15 frames/sec. Representative images are shown in Fig. 8B.

Supplementary Video S3. Dynamics of UPIb/UPIIIa-EGFP in MDCK cells where the AFs were depolymerized with cytochalasin D. Time-lapse movie of MDCK cells with depolarized AFs and expressing UPIb/UPIIIa-EGFP (green). Time-lapse confocal images were taken every 3.8 s for 8.5 min using a laser-scanning confocal microscope (LSM710; Carl Zeiss). Display rate is 15 frames/sec. Representative images are shown in Fig. 8C.
